# Perlidae (Plecoptera) from the Paranapiacaba Mountains, Atlantic Forest, Brazil: Diversity and implications of the integrative approach and teneral specimens on taxonomy

**DOI:** 10.1371/journal.pone.0243393

**Published:** 2020-12-10

**Authors:** Lucas Henrique de Almeida, Pitágoras da Conceição Bispo

**Affiliations:** 1 Programa de Pós-Graduação em Entomologia, Faculdade de Filosofia, Ciências e Letras de Ribeirão Preto, Universidade de São Paulo, Ribeirão Preto, São Paulo, Brazil; 2 Laboratório de Biologia Aquática, Departamento de Ciências Biológicas, Faculdade de Ciências e Letras de Assis, Universidade Estadual Paulista, Assis, São Paulo, Brazil; Laboratoire de Biologie du Développement de Villefranche-sur-Mer, FRANCE

## Abstract

The study of complementary sources of biological variation (e.g. morphological, molecular) has allowed a better understanding of biodiversity through the construction of an integrative taxonomy. Using this approach, specimens from the Paranapiacaba Mountains, southeastern Brazil, were studied to update the knowledge on the stonefly family Perlidae from the region, characterize the species, and make associations between nymphs and adults using a fragment of the *mitochondrial cytochrome c oxidase subunit I* (COI) gene. The study also discusses the implications of integrative taxonomy and teneral specimens for the study of South American Perlidae. The molecular data were analyzed using Bayesian inference, Neighbor-joining, and delimiting species methods. Our results revealed that, in general, there was a morphological and molecular congruence between species. In the Paranapiacaba Mountains, three genera and 15 species were recorded: *Anacroneuria boraceiensis* Froehlich 2004, *A*. *debilis* (Pictet 1841) (new record), *A*. *fiorentini* De Ribeiro and Froehlich 2007 (new record), *A*. *flintorum* Froehlich 2002, *A*. *iporanga* Bispo and Froehlich 2004, *A*. *itajaimirim* Bispo and Froehlich 2004, *A*. *polita* (Burmeister 1913), *A*. *subcostalis* Klapálek 1921, *A*. *tupi* Bispo and Froehlich 2004 (with a description of the nymph), *Kempnyia auberti* Froehlich 1996, *K*. *colossica* (Navás 1934), *K*. *flava* Klapálek 1916, *K*. *neotropica* (Jacobson and Bianchi 1905) (including its new junior synonym *K*. *petersorum* Froehlich 1996), *K*empnyia sp., and *Macrogynoplax veneranda* Froehlich 1984. COI sequences were obtained for 11 species, five of which had nymphs associated with adults. Among the five associated nymphs, the nymph of *A*. *tupi* is described here. The results of this study indicate that the color of adult teneral specimens differs from that of mature specimens. Given this, the synonym of *K*. *neotropica* and *K*. *petersorum* was proposed since these species have high morphological and molecular similarities and differ only in color patterns. In addition, the previous record of *A*. *petersi* Froehlich 2002 from the Paranapiacaba Mountains was invalidated since it was considered a teneral specimen of *A*. *flintorum*. These results suggest that the development of an integrative taxonomy is essential to continue advancing the study of Perlidae diversity in South America.

## Introduction

There are about 3700 species of stoneflies (Plecoptera) described in 16 families [1], among them about 500 species (six families) are found in South America [2]. In Brazil, there are about 200 species in eight genera and two families (Gripopterygidae and Perlidae) [2, 3]. Gripopterygidae is represented by *Gripopteryx* (Pictet 1841) [4], *Paragripopteryx* Enderlein 1909 [5], *Tupiperla* Froehlich 1969 [6], and *Guaranyperla* Froehlich 2001 [7], while Perlidae is represented by *Anacroneuria* Klapálek 1909 [8], *Enderleina* Jewett 1960 [9], *Kempnyia* Klapálek 1914 [10], and *Macrogynoplax* Enderlein 1909 [5]. Within Perlidae, *Anacroneuria* has been recorded from the southern United States to northern Argentina [11] and is the most diverse genus of the family, with more than 380 species described [2], more than 80 of which are from Brazil. *Enderleina* is distributed in Venezuela and north of Brazil and includes nine species, with seven from Brazil [11, 12]. *Kempnyia* currently includes 37 species, is endemic to Brazil, and is found in the mountains from Central Brazil and Bahia State to Rio Grande do Sul State [11, 13–15]. *Macrogynoplax*, which has been recorded from northern South America (Suriname, Guyana, Venezuela, Peru, northern Brazil) to the southeast of Brazil, includes 16 species, eight of which are in Brazil [11, 16, 17].

The diversity of Brazilian perlids has been consistently studied since the 1980s [18–20]. However, despite the relevant growth in knowledge about Perlidae, in general, most studies have been restricted to the morphology of adults, mainly males. In fact, few nymphs have been associated with adults and described [21]. The nymphs of stoneflies live mainly in streams and are important indicators of environmental quality because, in general, they require well-oxygenated clean water to survive [22]. These requirements contribute to make nymph rearing in the laboratory and subsequent association with adults difficult [23, 24]. Although some authors have successfully associated Neotropical nymphs with adults [16, 22, 25–34], finding a sufficient number of nymphs, keeping them alive during transport, in a laboratory, or in an artificial stream, and feeding them until emergence are challenges that need to be considered. Nymph rearing strategies are very time consuming and can hinder the nymph-adult association, mainly for rare species with narrow environmental tolerance, creating difficulties for a broad research program aimed at describing nymphs. In Brazil, many nymphs that have been described were associated with adults by rearing in the laboratory [16, 23, 26, 35] or in streams in the field [16, 22, 25, 26, 33, 36, 37], and few species were associated using molecular tools [21, 24, 38]. Despite these efforts, currently, only 28 of the 145 species of Brazilian perlids have been associated and described. Knowledge of nymphs is essential for obtaining useful characters for proposing morphology-based phylogenies and for ecological and biomonitoring studies, as they experience environmental changes in water.

In addition to the associated life stages (e.g. adults and nymphs), other obstacles also need to be overcome to better understand the diversity of Plecoptera, including the understanding of intraspecific variability (intra and inter-population) since most species have been described based on a few individuals [39–43]. One solution to overcome these obstacles is to expand the sampling effort, mainly in poorly sampled areas, and seek to develop an integrative taxonomic approach that considers both morphological and molecular evidence [44–46]. Furthermore, the use of an integrative approach could help to solve the problem of delimiting several closely related Perlidae species since some variations/species are difficult to delimit using only morphological adult male characters. In addition to morphology, molecular tools have been used successfully to study stoneflies around the world, mainly to associate life stages [47–49], identify cryptic species problems [50], delimit species [51], and address phylogenetic and biogeographic questions [52–59]. In Brazil, molecular tools have been mainly used to successfully associate the nymphs of Plecoptera [21, 24, 38]. However, other issues also need to be addressed, including the understanding of the molecular identity and intraspecific variability and their congruences with each morphological-based species of Plecoptera.

Perlids from the Intervales State Park (São Paulo State, Brazil) in the Paranapiacaba Mountains were studied by Bispo and Froehlich [60]. They morphologically characterized the specimens from that region and recorded 14 species from three genera of Perlidae (*Anacroneuria*, eight species; *Kempnyia*, five species; and *Macrogynoplax*, one species). Here, the study of Bispo and Froehlich [60] is revisited, integrating morphological and molecular data, as well as expanding the number of specimens studied and the conservation areas sampled. The present study was carried out with the following objectives: 1) to update the knowledge of Perlidae from the Paranapiacaba Mountains, using both morphological and molecular data; 2) to characterize species and make associations between nymphs and adults based on the barcode region of the *mitochondrial cytochrome c oxidase subunit I* (COI) gene [61, 62]; 3) to assess the implications of intraspecific and interspecific COI divergence to delimit species; and 4) to assess the implications of teneral patterns on the taxonomy of Perlidae. The findings obtained here can influence how we study the diversity of Perlidae in Brazil and South America in the future.

## Methods

All specimens were collected according to Brazilian law, including collection licenses from Sistema de Autorização e Informação em Biodiversidade (SISBIO– 55428–9) and Comissão Técnico-Científica do Instituto Florestal (COTEC– 260108–008.106/2016). In addition, animal handling was carried out according to the normative instructions of the Instituto Chico Mendes de Conservação da Biodiversidade (ICMBio).

**Fig 1:**
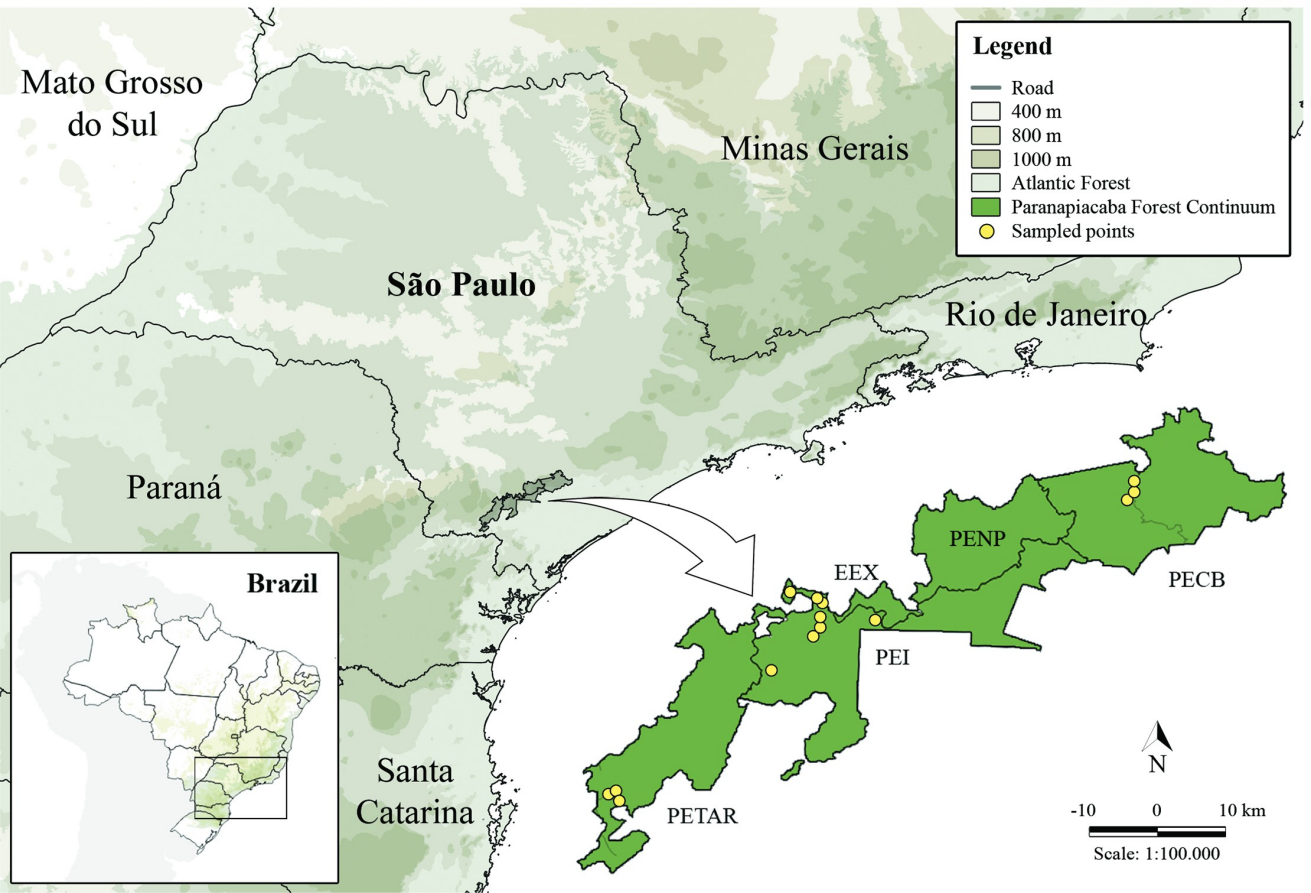
Paranapiacaba Mountains. Map of Paranapiacaba Forest Continuum and the sampled sites. EEX: Ecological Station of Xitué; PECB: Carlos Botelho State Park; PEI: Intervales State Park; PENP: Nascentes do Paranapanema State Park; and PETAR: Alto Ribeira Tourist State Park, São Paulo State, Brazil.

The specimens of Perlidae studied were sampled in the Paranapiacaba Mountains, whose biodiversity is protected by five conservation areas: 1) Ecological Station of Xitué; 2) Carlos Botelho State Park (PECB); 3) Intervales State Park (PEI); 4) Nascentes do Paranapanema State Park; and 5) Alto Ribeira Tourist State Park (PETAR), São Paulo State, Brazil (Fig 1) [63]. These conservation areas form the Paranapiacaba Forest Continuum, one of the least disturbed areas of the Atlantic Forest in Brazil, amounting to 1200 km^2^ and having an altitude ranging from 30 to 1200 m [63]. The Paranapiacaba Forest Continuum also incorporates the headwaters of two important hydrographic basins (Alto Paranapanema and Alto Ribeira basins) [63] and is relevant for conserving aquatic insects from mountain streams [64].

We sampled Plecoptera specimens in streams with cool and well-oxygenated clean waters in PECB, PEI, and PETAR. The adults were attracted using the both the traditional white sheet and light pan traps, with both white and ultraviolet lamps [65, 66]. Nymphs were actively sampled using a D-net in pools and riffles. The specimens collected were housed in the Aquatic Biology Laboratory Collection (UNESP, Assis). The material was preserved in 80% ethanol in an air-conditioned room (17°C) or in absolute ethanol at -20°C. We used absolute ethanol for material collected in recent years to preserve the quality of DNA for molecular analyses. Most specimens were collected by Lucas Henrique de Almeida (LHA), Pitágoras da Conceição Bispo (PCB), Claudio Gilberto Froehlich (CGF), Adriano Sanches Melo (ASM), Henrique Paprocki (HP), Lucas Silveira Lecci (LSL), and Vera Lucia Crisci-Bispo (VLCB).

We re-studied 54 of the 84 males studied by Bispo and Froehlich [60]. In addition, we studied new material (58 males, 25 females, and 14 nymphs) from our fieldwork. All types studied are deposited at the Museum of Zoology of the University of São Paulo (MZUSP). The paratype of *Anacroneuria petersi* Froehlich 2004 [67] was studied to compare with a specimen previously identified in PEI [67]. Five specimens of *Kempnyia colossica* (Navás 1934) [68] from two other locations (Morretes, Paraná State and Idaial, Santa Catarina State) were included in the analyses in an attempt to better understand the morphological variation exhibited by species.

For identification, the abdomens of all adult males were severed between segments seven and eight and placed in microtubes containing potassium hydroxide (10%) overnight to clear the tissues around the penial armature. Later, the penial armatures were transferred to new microtubes containing acetic acid to neutralize the reaction. Specimens were identified based on studies of Zwick [69], Froehlich [20, 67, 70], Bispo and Froehlich [33, 60], and Bispo *et al*. [36]. Nymph descriptions were carried out based on the model followed by Bispo and Froehlich [33] and Bispo *et al*. [36]. Illustrations of cleaned penial armatures and subgenital plates were made using a lucida camera mounted on a Leica DM1000 microscope and rendered in Adobe Illustrator CS6® editor. All images were taken using a digital camera (Leica DFC450) on a Leica M205A stereomicroscope and optimized in Adobe Photoshop CS3® editor.

Total DNA was extracted from the specimens using the DNeasy® Blood and Tissue Kit (Qiagen) following the manufacturer’s protocol. DNA was extracted from at least three specimens of each identified species and nymph morphotypes to increase the likelihood of association. When this number was not reached, DNA was extracted from the available specimens. One leg was used, and each specimen received a separate catalog number (S1 Table) to link the sequences with the specimen. Using polymerase chain reaction (PCR), the barcode region of the COI gene [61, 62] was amplified using primers LCO-1490 (5'-GGTCAACAAATCATAAAGATATTGG-3') and HCO-2198 (5'-TAAACTTCAGGGTGACCAAAAAATCA-3') [71]. We used a PCR program with a first denaturation step at 94°C (2 minutes), followed by 40 cycles of denaturation at 94°C (1 minute), annealing at 50°C (1 minute), and extension at 72°C (2 minutes), and a final extension step at 72°C (5 minutes). Purification and bidirectional sequencing of the products were performed by Helixxa (a Brazilian company specializing in biotechnology).

Using MEGA 7 [72], we manually edited the chromatograms to obtain consensus sequences and aligned them using ClustalW [73]. A 636-base pair (bp) alignment of 54 sequences was obtained, with 25 for *Anacroneuria*, 24 for *Kempnyia*, four for *Macrogynoplax*, and one for *Gripopteryx* (Outgroup). Bayesian analysis was performed using MrBayes 3.2.2 [74] (2 independent runs of 4 Monte Carlo-Markov Chains for 1250000 generations, 25% generation burn-in) and Neighbor-Joining analysis using MEGA 7 [72] (1000 bootstrap replicates). For Bayesian analysis, Partitionfinder 2.1.1 [75] was used to choose the best evolutionary models. The chosen models were GTR+G, HKY+I, and GTR+I+G for the first, second, and third positions of the codons. For Neighbor-joining analysis, the pairwise distances were calculated using the Kimura-2-parameter (K2P). To assess species delimitation based on molecular data, the following methods were used: Automatic Barcode Gap Discovery (ABGD), considering primary (ABGD_p_), and recursive (ABGD_r_) partitions [76]; Poisson Tree Processes (PTP); and Bayesian implementation of the Poisson Tree Processes (bPTP) [77]. These analyses were performed using online servers (ABGD, https://bioinfo.mnhn.fr/abi/public/abgd/; and PTP/bPTP, http://species.h-its.org/ptp/). For ABGD analyses, K2P distances with default parameters and a relative gap width of 1.0 were used. For PTP and bPTP models, the tree resulting from Bayesian analysis and default settings were used. All COI sequences used in this study are available on GenBank (S1 Table).

## Results

We found 15 species, including a new synonym, in three genera of Perlidae in the Paranapiacaba Mountains (Table 1). The barcode region of the COI gene from 11 of these species was amplified (Table 1). For the first time since its description, the COI of *Kempnyia petersorum* Froehlich 1996 [70] from its type location was sequenced to assess the hypothesis that this species is synonymous with *K*. *neotropica* (Jacobson and Bianchi 1905) [78]. As one of the results of our integrative approach, an updated key for the identification of Perlidae adult males from the Paranapiacaba Mountains is presented (S1 Appendix).

**Table 1:** Perlidae species recorded in Paranapiacaba Mountains (São Paulo State, Brazil) with known life stages and distribution.

Species	Species description	Nymph description	Distribution**	COI
*Anacroneuria boraceiensis* Froehlich 2004	[67]	-	MG, SP	No
*Anacroneuria debilis* (Pictet 1841)*	[4]	[35]	Argentina, Paraguay. BA, ES, GO, MG, PR, RJ, RO, RS, SC, SE, SP, TO	Yes
*Anacroneuria fiorentini* De Ribeiro and Froehlich 2007*	[79]	-	RS, SC, SP*	No
*Anacroneuria flintorum* Froehlich 2002	[80]	[24]	ES, MG, RJ, RS, SC, SP	Yes
*Anacroneuria iporanga* Bispo and Froehlich 2004	[60]	[22]	SP	No
*Anacroneuria itajaimirim* Bispo and Froehlich 2004	[60]	-	SP	Yes
*Anacroneuria polita* (Burmeister 1839)	[81]	-	Argentina. MG, PR, SC, SP	Yes
*Anacroneuria subcostalis* Klapálek 1921	[82]	-	ES, RJ, SP	Yes
*Anacroneuria tupi* Bispo and Froehlich 2004	[60]	This paper	SP	Yes
*Kempnyia auberti* Froehlich 1996	[70]	-	PR, SP	No
*Kempnyia colossica* (Navás 1934)	[68]	[36]	MG, PR, RJ, SC, SP	Yes
*Kempnyia flava* Klapálek 1916	[83]	-	ES, RJ, SP	Yes
^*1*^*Kempnyia neotropica* (Jacobson and Bianchi 1905)	[78]	[33]	BA, ES, GO, MG, PR, RJ, RS, SC, SP	Yes
^*2*^*Kempnyia petersorum* Froehlich 1996	[70]	[21]	ES, MG, PR, RJ, SP	Yes
*Kempnyia* sp.	-	-	SP	Yes
*Macrogynoplax veneranda* Froehlich 1984	[19]	[19]	ES, SP	Yes

In this paper, the species ^1,2^ will be considered synonymous (see “Taxonomy” section).

*new record

**abbreviations of the Brazilian states based on national standard (S2 Fig).

### Molecular variation and species delimitation

**Fig 2:**
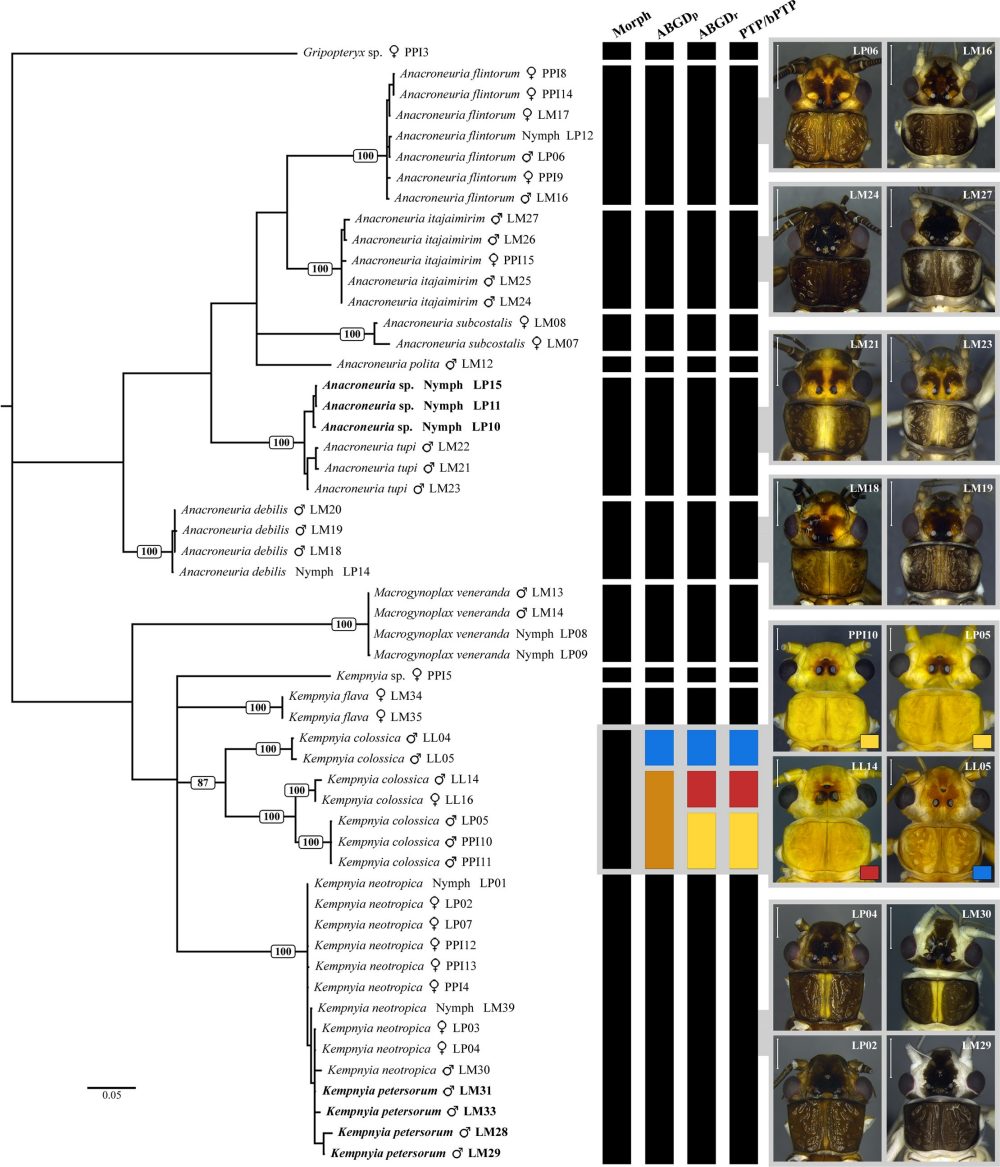
Molecular analysis. Bayesian inference tree obtained using *mitochondrial cytochrome c oxidase subunit I* (COI) sequences (636 bp) from specimens from the Paranapiacaba Mountains (São Paulo State, Brazil) and related stoneflies from Paraná State (red rectangle) and Santa Catarina State (blue rectangle), Brazil. Numbers are clusters support (bootstrap) recovered. The tree was rooted using a sequence of Gripopterygidae. Delimitation methods: 1) morphology (Morph); 2) Automatic Barcode Gap Discovery (ABGD) considering primary partition (ABGDp); 3) ABGD considering recursive partition (ABGDr); and 4) Poisson Tree Processes (PTP) and Bayesian implementation of the Poisson Tree Processes (bPTP).

Visual inspection of the COI trees (Bayesian and Neighbor-joining) and the results of delimiting species methods (ABGD, PTP, and bPTP) revealed that the clusters of the specimens were mostly consistent with the species previously defined by morphology (Fig 2). However, our analyses suggested that *K*. *neotropica*, and *K*. *petersorum* are the same species, and *K*. *colossica* is at least two species (Fig 2, S1 Fig). In the case of *K*. *colossica*, which was sampled from three different locations, ABGD_p_ suggested two species, and ABGD_r_, PTP, and bPTP suggested three (Fig 2).

The intraspecific variation considering only specimens from the Paranapiacaba Mountains was less than 4%. In the case of *Anacroneuria*, the following intraspecific divergences were found: *A*. *flintorum* ranged from 0 to 0.8%; *A*. *itajaimirim* from 0 to 1.1%; *A*. *tupi* from 0 to 2.3%; *A*. *subcostalis* 1.9%; and *A*. *debilis* from 0 to 0.3% (S2 Table). For *Kempnyia*, the following intraspecific divergences were found: *K*. *colossica* only from the Paranapiaca Mountains had 0% of divergence; *K*. *flava* also had 0%; and the cluster formed by *K*. *neotropica* + *K*. *petersorum* had a divergence ranging from 0 to 3.6% (S2 Table). Notably, although *K*. *colossica* did not have intraspecific variation when only specimens from the Paranapiacana Mountains were considered, this variation increased to 5.6% when specimens from Paraná State were included and to 13.9% when including specimens from Santa Catarina State. In the case of *Macrogynoplax veneranda*, intraspecific divergence ranged from 0 to 0.2% (S2 Table).

**Fig 3:**
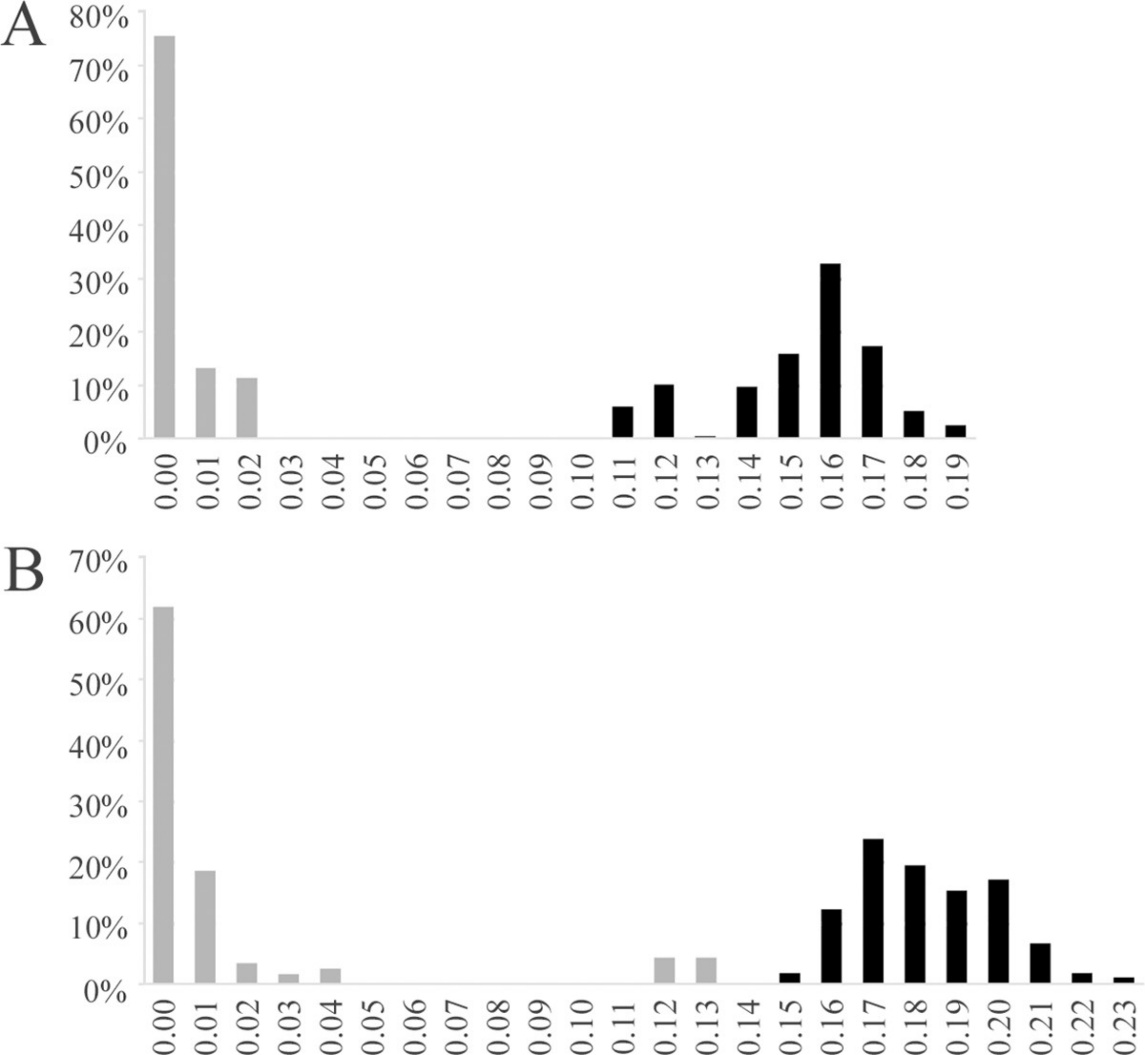
Intra- and interspecific divergences comparisons. Percentages of pairwise intra- and interspecific divergences obtained by Kimura-2-parameter (K2P) showing the barcode gap in *Anacroneuria* (A) and *Kempnyia* (B).

The frequency distribution of intraspecific and interspecific pairwise distances (K2P) revealed a barcode gap both for *Anacroneuria* and *Kempnyia* species (Fig 3). Interspecific variations were always greater than 11%. For all *Anacroneuria* species studied, interspecific divergence ranged from 11.3% between *A*. *itajaimirim* (LM26) and *A*. *polita* (LM12) to 19.2% between *A*. *subcostalis* (LM07) and *A*. *tupi* (LP10). For *Kempnyia*, interspecific divergence ranged from 15.3% between *K*. *petersorum* (LM30) and *K*. *flava* (LM34) to 23.1% between *K*. *petersorum* (LM28) and *K*. *colossica* (LP05 and LL14). Notably, the lowest interspecific divergence found in *Kempnyia* was 15.3%, which is close to the highest intraspecific divergence found for *K*. *colossica*, considering all specimens studied here. According to species delimitation methods, *K*. *colossica* is probably a complex of species.

#### Association of the nymphs and variation in color patterns of the adults

Nymphs of five species were associated with adults using COI sequences (Fig 2): *Anacroneuria flintorum*, *A*. *debilis*, *A*. *tupi*, *Kempnyia colossica*, *K*. *neotropica*, and *Macrogynoplax veneranda*. Here, we described the nymph of *A*. *tupi*, since the other four associated nymphs have already been described [19, 24, 33, 35, 36].

**Fig 4:**
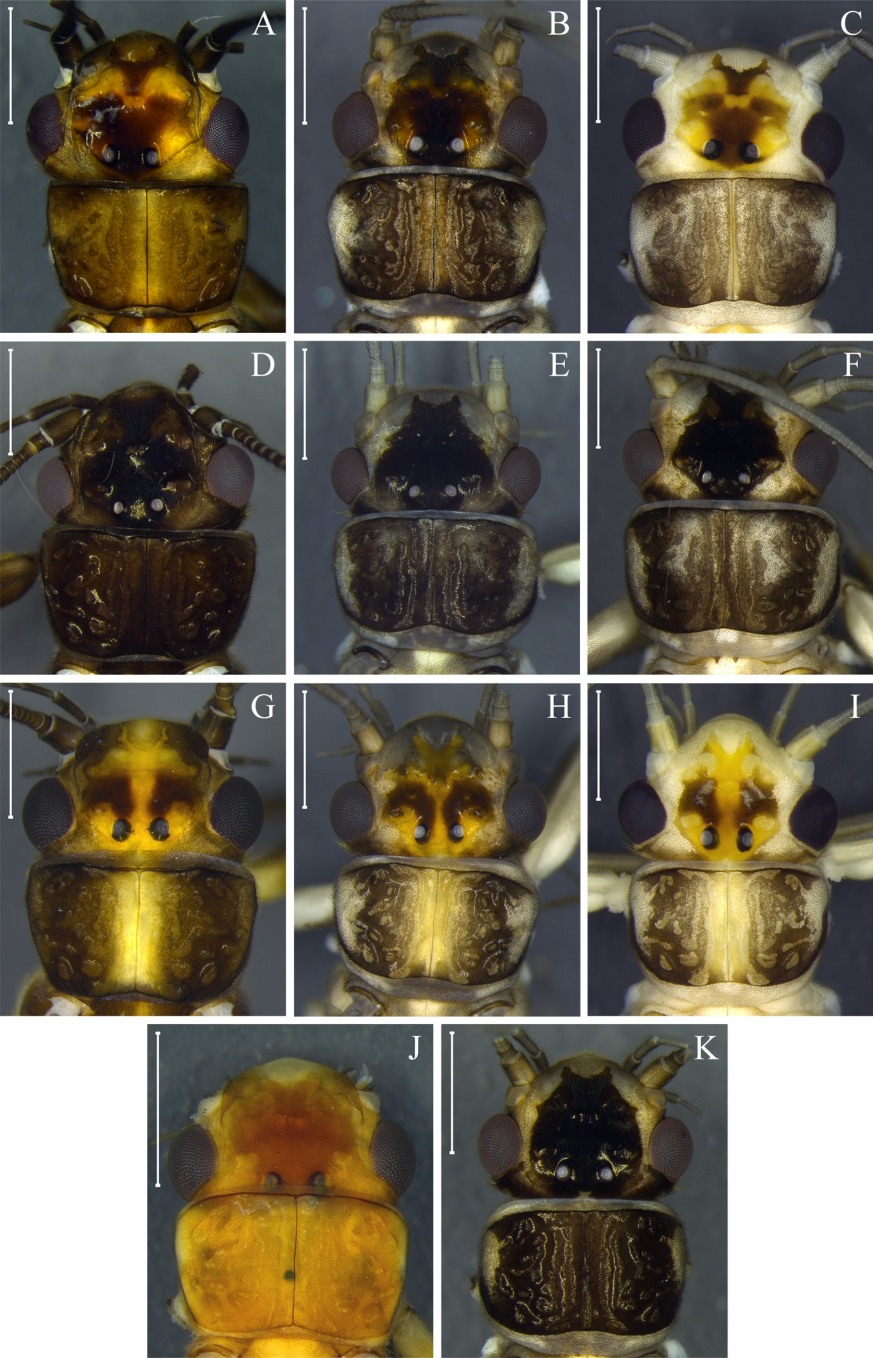
Teneral pattern of *Anacroneuria* species. Head and pronotum of *A*. *debilis* adult male (A) and teneral adult males (B and C); *A*. *itajaimirim* adult male (D) and teneral adult males (E and F); *A*. *tupi* adult female (G) and teneral adult males (H and I); *A*. *polita* adult female (J) and teneral adult male (K). Scale: 1 mm.

**Fig 5:**
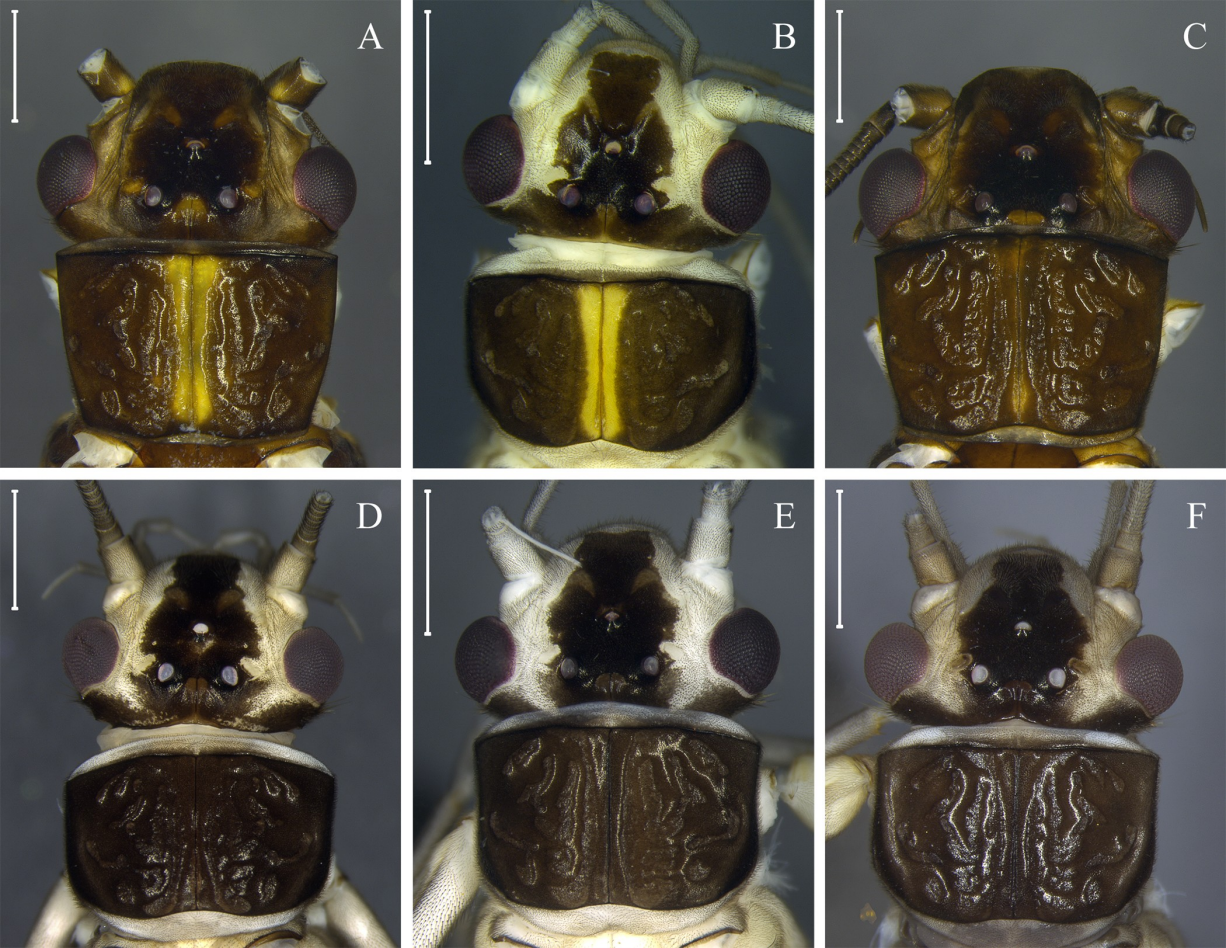
Morphological variation within cluster *Kempnyia neotropica* + *K*. *petersorum*. Head and pronotum of adult females (A and C) and teneral adult female (B) of *K*. *neotropica*. Head and pronotum of adult males (D–F) of *K*. *petersorum* according to description by Froehlich [70]. Scale: 1 mm.

Our Bayesian and Neighbor-joining analysis revealed that adults of the same species had variations in color pattern (Fig 2, S1 Fig). This variation could be related to the presence of teneral specimens, which in Perlidae can be identified by the presence of remnants of thoracic gills. In general, teneral specimens present both variation in morphology (rounded pronotum without lateral fold) and color pattern (milky wings, and white to grayish coloration on part of the body, mainly around the head) [24]. The variation in color is more remarkable when considering dark species. Here, it was possible to observe color pattern variations between teneral and mature adults in *Anacroneuria flintorum*, *A*. *debilis*, *A*. *itajaimirim*, *A*. *tupi*, *A*. *polita* (Figs 2 and 4), and in cluster *K*. *neotropica* + *K*. *petersorum* (Figs 2 and 5). As some species recorded in the Paranapiacaba Mountains were identified or described based on morphological characters and color patterns of teneral specimens, some adjustments were necessary, which will be presented in the "Taxonomy" section.

In addition to the variation in color pattern as a function of time since adult emergence, it is also important to consider that there may be fading due to the storage time of the specimen in ethanol, which was also observed in the present study. However, these two phenomena can be separated from each other since the specimens stored for a long time in ethanol are homogeneously less pigmented than recently collected specimens, and the teneral specimens, in general, have pale and milky colors on specific parts of the body (e.g., lateral part of the head and wings), as well as remaining gills [24].

### Taxonomy

#### *Anacroneuria boraceiensis* Froehlich 2004

**Fig 6:**
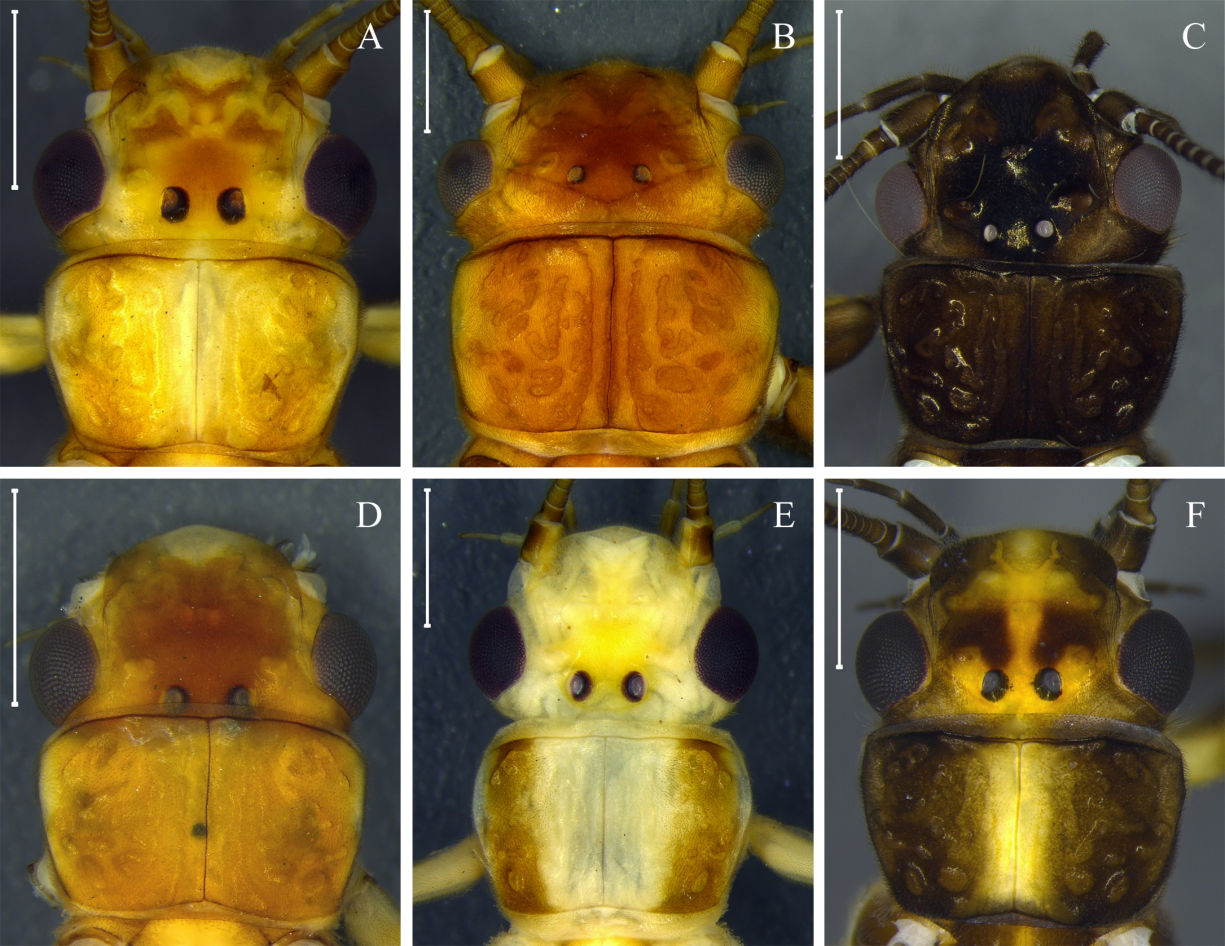
*Anacroneuria* species. Head and pronotum of *A*. *boraceiensis* adult male (A), *A*. *iporanga* adult male (B), *A*. *itajaimirim* adult male (C), *A*. *polita* adult male (D), *A*. *subcostalis* adult female (E), and *A*. *tupi* adult male (F). Scale: 1 mm.

*Anacroneuria boraceiensis* Froehlich 2004: 54, description [67]; Bispo and Froehlich 2004: 103, record [60]; Froehlich 2010: 149, catalog [3]; Novaes and Bispo 2014: 434, record [42]. (Fig 6A)

*Material examined*. **BR, SP, Iporanga**: PEI, Rio Poços Altos, 24°18’13”S, 48°20’30”W, 08.xi.1992, 1 male; 18.xi.1992, CGF col., 1 male; 10.xi.1993, CGF and HP col., 1 male and 1 female.

*Morphometric data*. Male (n = 3) forewing length: 10.9–11.3 mm. Female (n = 1) forewing length: 14 mm.

*Remarks*. The male’s penial armature of *A*. *boraceiensis* [67] slightly resemble that of *A*. *amargosa* Righi-Cavallaro and Froehlich 2013 [40]. However, *A*. *boraceiensis* is larger (*A*. *amargosa*: 8.0–9.5 mm) and has a distinctive head color pattern (Fig 6A) [40, 67].

#### *Anacroneuria debilis* (Pictet 1841)

**Fig 7:**
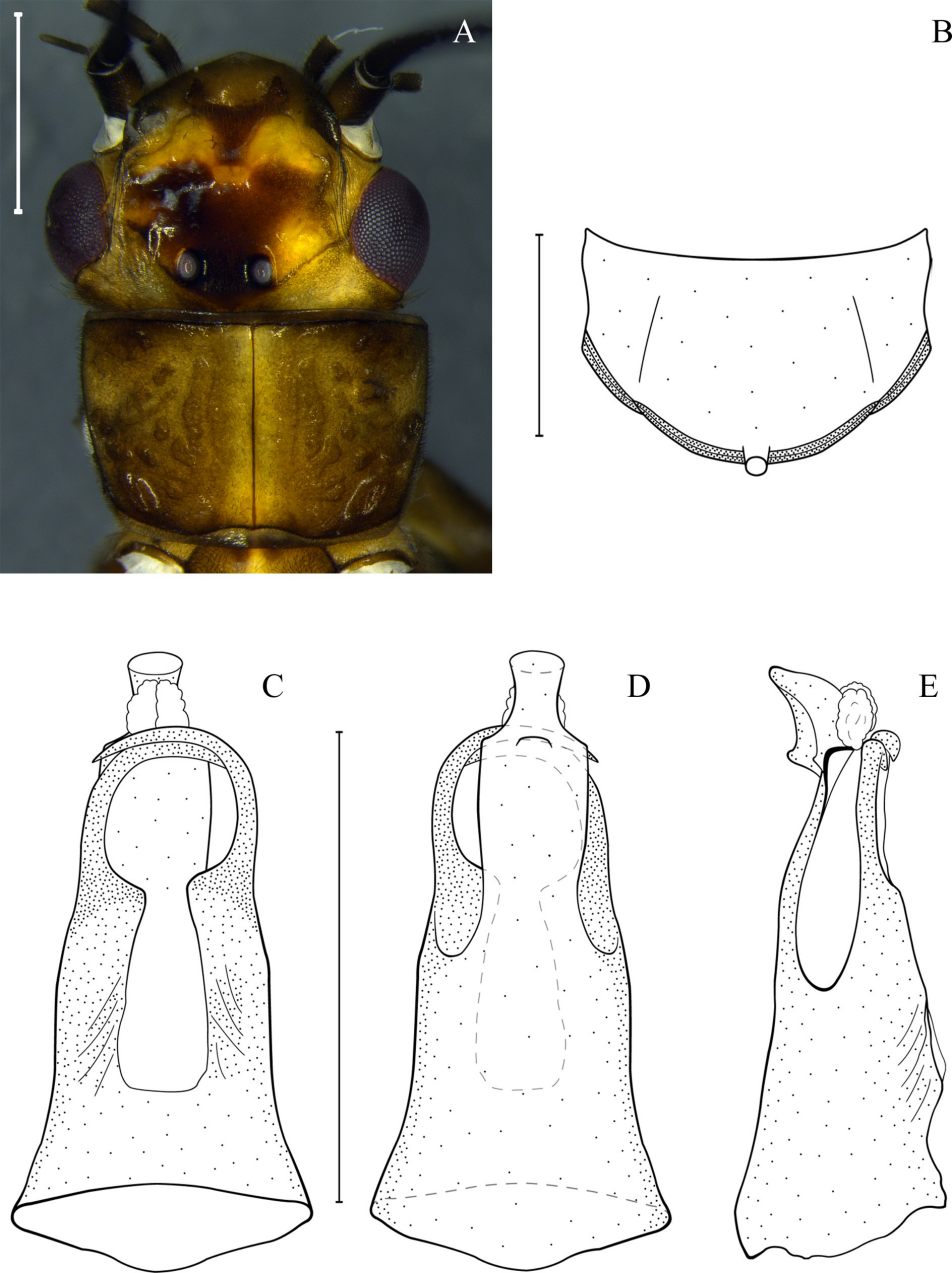
*Anacroneuria debilis*. Adult male, head and pronotum (A). Tergum 9 with hammer (B). Penial armature in ventral (C), dorsal (D), and lateral (E) views. Scale: 1 mm.

*Perla* (*Perla*) *debilis* Pictet 1841: 255, description [4]; *Anacroneuria debilis* Zwick 1972: 1155, holotype illustration [69]; Zwick 1973: 486, illustration [84]; Froehlich 2002: 76, illustration [80]; Froehlich 2010: 154, catalog [3]; Froehlich 2010: 56, record [85]; Avelino-Capistrano *et al*. 2011: 60, nymph description [35]; Baldin *et al*. 2013: 392, illustration [39]; Bispo *et al*. 2014: 592, illustration and picture [41]; Novaes and Bispo 2014: 459, illustration and picture [86]; Novaes and Bispo 2014: 434, record [42]; Duarte and Lecci 2016: 293, record [87]; Novaes *et al*. 2016: 95, record [88]; Almeida and Duarte 2017: 481, picture [89]; Gonçalves *et al*. 2017: 146, record [43]; Rippel *et al*. 2019: 359, record [90].

(Figs 4A–4C and 7A–7E)

*Material examined*. **BR, SP, Iporanga**: PEI, Ribeirão Água Comprida, 24°17’38”S, 48°25’04”W, 27.x.1999, PCB and VLCB col., 1 male; PEI, Rio do Carmo, bridge, 24°18’15”S, 48°24’31”W, 28.x.1999, PCB and VLCB col., 7 males; 30.x.2002, 1 male; PEI, Rio do Carmo, near Córrego do Inferno, 24°18’15”S, 48°24’31”W, 29.ix.2002, ASM col., 1 male; PEI, Ribeirão Lajeado, 24°18’15”S, 48°24’39”W, 14.xii.2014, PCB *et al*. col., 1 nymph; 10.ii.2017, LHA *et al*. col., 4 males and 2 nymphs. **SP, Apiaí**: PETAR, Núcleo Ouro Grosso, Rio Betari, 24°32'45"S, 48°40'59"W, 12.ii.2017, LHA *et al*. col., 1 male; PETAR, Núcleo Santana, Rio Roncador, 24°32’00”S, 48°42’06”W, 15.ii.2017, LHA *et al*. col., 1 male.

*Morphometric data*. Male (n = 16) forewing length: 10.0–11.4 mm.

*Remarks*. This is the first record of this species in the Paranapiacaba Mountains. The specimens studied (Fig 7A) present high similarity in size and color pattern with *A*. *flintorum*. Teneral and mature specimens exhibit a similar pattern of brown and yellow spots near the M line (Fig 4A–4C). The penial armature of the specimens studied agrees with the holotype illustration [69] and is similar to that illustrated by Novaes and Bispo [86] (Fig 7C–7E). However, the penial armature of our specimens are slightly different from that illustrated by Baldin *et al*. [39] and Bispo *et al*. [41]. Our specimens have a dorsally pointed apex from the lateral view, while those mentioned point upwards [39, 41].

#### *Anacroneuria fiorentini* De Ribeiro and Froehlich 2007

**Fig 8:**
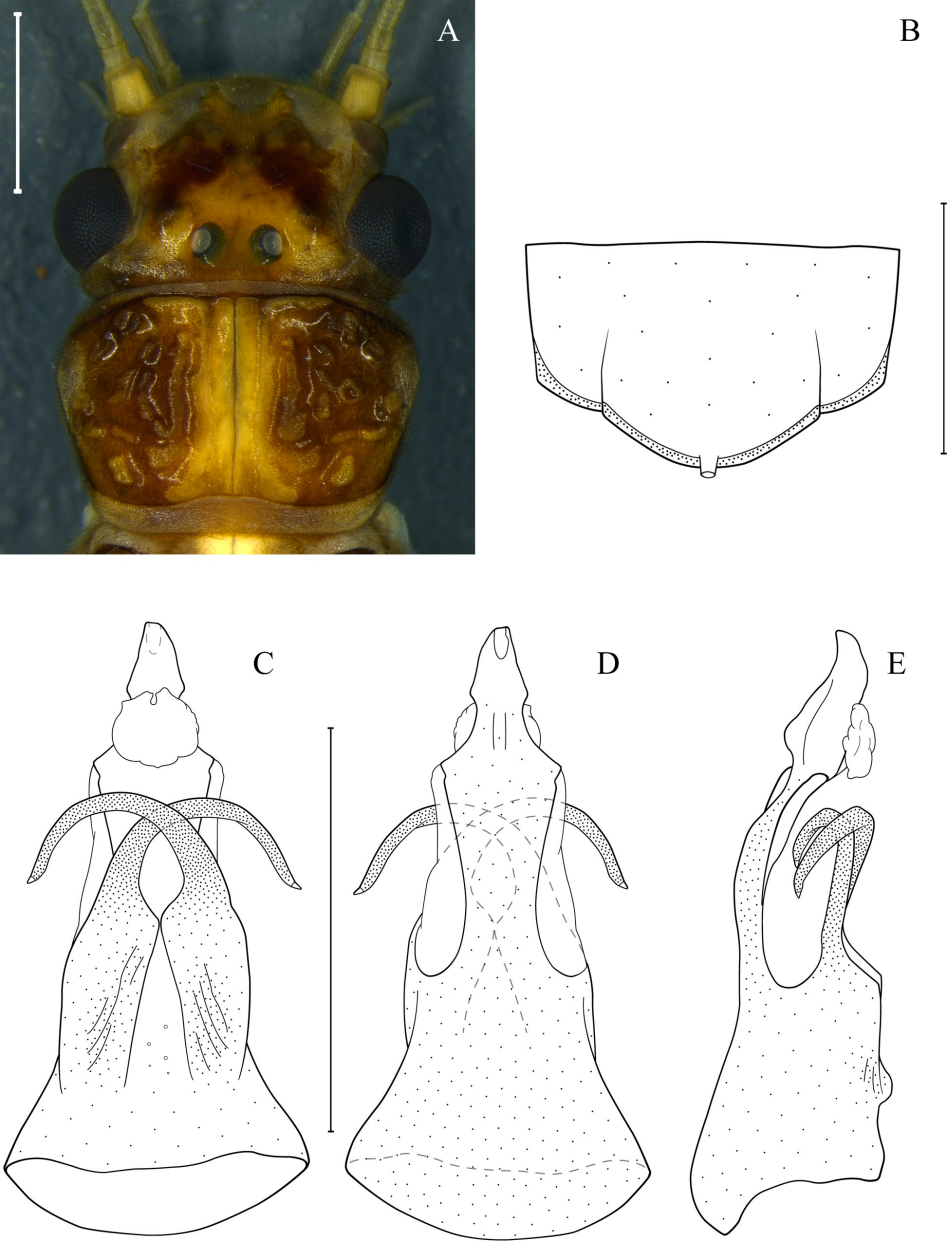
*Anacroneuria fiorentini*. Teneral male, head and pronotum (A). Tergum 9 with hammer (B). Penial armature in ventral (C), dorsal (D), and lateral (E) views. Scale: 1 mm.

*Anacroneuria fiorentini* De Ribeiro and Froehlich 2007: 53, description [79]; Froehlich 2010: 155, catalog [3]; Novaes and Bispo 2014: 278, illustration and picture [91]. (Fig 8A–8E)

*Material examined*. **BR, SP, Iporanga**: PEI, Rio do Carmo (Base Alecrim), 11.viii.2010, LSL col., 1 male.

*Morphometric data*. Male (n = 1) forewing length: 11.1 mm.

*Remarks*. This is the first record of this species in São Paulo State. The specimen collected is a teneral and, therefore, the color pattern presented here should be viewed with caution. The specimen present a pale body, almost complete pronotum folds, remnants of gills on the thoracic segments, and milky wings (Fig 8A) [24]. Although teneral, this specimen agrees with the description by De Ribeiro and Froehlich [79], both in the disposition of brown spots on the head and color pattern of the pronotum but present slight variation in penial armature. From the lateral view, the penial armature is similar, but the apex is less inclined dorsally (Fig 8E).

#### *Anacroneuria flintorum* Froehlich 2002

*Anacroneuria flintorum* Froehlich 2002: 93, description [80]; Bispo and Froehlich 2004: 99, female description and male illustration [60]; De Ribeiro and Froehlich 2007: 55, record [79]; Froehlich 2010: 156, catalog [3]; Froehlich 2010: 56, illustration [85]; Baldin *et al*. 2013: 394, record [39]; Gonçalves *et al*. 2017: 146, record [43]; Almeida *et al*. 2018: 413, distribution, nymph description and male picture [24].

*Material examined*. **BR, SP, São Miguel Arcanjo**: PECB, Rio Taquaral, bridge, 24°03’31”S, 47°59’41”W, 09.ix.2006, PCB col., 1 male; 05.ii.2017, LHA *et al*. col., 1 nymph; PECB, Ribeirão de Pedras, bridge, 24°03’40”S, 47°59’51”W, 06.ii.2017, LHA *et al*. col., 1 nymph. **SP, Iporanga**: Gruta da Tapagem, 24°38’09”S, 48°24’13”W, 15.xi.1991, 1 male; PEI, Rio do Carmo, bridge, 19.ii.1992, CGF col., 1 male; 16.ii.1993, CGF col., 2 males; 28.x.1999, PCB and VLCB col., 1 male; 25.iii.2000, PCB and VLCB col., 1 male; 14.xii.2014, PCB *et al*. col., 1 male and 1 female; 08.ii.2017, LHA *et al*. col., 1 male; PEI, Rio Poços Altos, 10.xi.1993, CGF and HP col., 2 males; 01.ii.2000, PCB and CGF col., 2 males; PEI, Ribeirão Lajeado, 24.ii.1999, 2 males; 17.ix.1999, PCB and VLCB col., 1 male; PEI, Ribeirão Água Comprida, 27.x.1999, PCB and VLCB col., 3 males; 23.xi.1999, PCB and VLCB col., 7 males; 06.i.2000, PCB and VLCB col., 8 males; 30.x.2002, ASM col., 1 male; PEI, Córrego Roda D’Água, 24°16’17”S, 48°25’20”W, 13.xii.2014, PCB *et al*. col., 1 female; PEI, Córrego do Mirante, 24°16’36”S, 48°24’56”W, 15.xii.2014, PCB *et al*. col., 1 female; PEI, Ribeirão Bocaina, bridge, 24°16’13”S, 48°27’09”W, 12.ii.2017, LHA *et al*. col., 1 male. **SP, Apiaí**: PETAR, Rio Betari, Bairro da Serra, 24°33’08”S, 48°40’36”W, 20.viii.1996, ASM col., 1 male; PETAR, Núcleo Santana, Riacho Furnas, 24°32’03”S, 48°42’02”W, 14.ii.2017, LHA *et al*. col., 2 males; PETAR, Núcleo Santana, Rio Roncador, 15.ii.2017, LHA *et al*. col., 1 male.

*Morphometric data*. Male (n = 41) forewing length: 10.0–11.5 mm. Female (n = 3) forewing length: 13.0–14.2 mm.

*Remarks*. *Anacroneuria flintorum* is the most frequently found species in the Paranapiacaba Mountains. In a previous study, Bispo and Froehlich [60] recorded *A*. *petersi* in the Paranapiacaba Mountains based on one male specimen. Based on the teneral pattern found by Almeida *et al*. [24] for *A*. *flintorum*, studying the paratype of *A*. *petersi*, and reanalyzing the penial armature of the specimen identified previously as *A*. *petersi* [60], it is now possible to conclude that this specimen is a teneral of *A*. *flintorum*. Therefore, here, the previous record of *A*. *petersi* in the Panapiacaba Mountains is invalidated.

#### *Anacroneuria iporanga* Bispo and Froehlich 2004

*Anacroneuria iporanga* Bispo and Froehlich 2004: 103, description [60]; Froehlich 2010: 158, catalog [3]; Almeida *et al*. 2019: 142, nymph description [22].

(Fig 6B)

*Material examined*. **BR, SP, Iporanga**: PEI, Ribeirão Água Comprida, 23.ix.1999, PCB and VLCB col., 1 male (Holotype/MZUSP); PEI, Ribeirão Bocaina, 28-30.xi.2000, PCB col., 1 male (Paratype/MZUSP).

*Morphometric data*. Male (n = 2) forewing length: 16–17 mm.

*Remarks*. This species was described by Bispo and Froehlich [60] and the nymph by Almeida *et al*. [22], both based on material from PEI. This species is brown (Fig 6B) and has a characteristic hammer and penial armature [60].

#### *Anacroneuria itajaimirim* Bispo and Froehlich 2004

*Anacroneuria itajaimirim* Bispo and Froehlich 2004: 105, description [60]; Froehlich 2010: 159, catalog [3].

(Figs 4D–4F and 6C)

*Material examined*. **BR, SP, Iporanga**: PEI, Ribeirão Bocaina, 21.i.2011, LSL col., 1 male; PEI, Córrego Roda D’Água, 16.ii.2013, 1 male; 13.xii.2014, PCB *et al*. col., 1 female; 07.ii.2017, LHA *et al*. col., 1 male; PEI, Rio do Carmo, bridge, 09.ii.2017, LHA *et al*. col., 2 males. **SP, Apiaí**: PETAR, Núcleo Ouro Grosso, Trilha das Figueiras, 24°32'43"S, 48°40'53"W, 13.ii.2017, LHA *et al*. col., 1 male; PETAR, Núcleo Santana, Riacho Furnas, 14.ii.2017, LHA *et al*. col., 4 males.

*Morphometric data*. Male (n = 11) forewing length: 10.8–11.5 mm. Female (n = 1) forewing length: 12.5 mm.

*Remarks*. This species has been recorded only in the Paranapiacaba Mountains [60]. This species is dark brown (Figs 4D and 6C) but can become ochraceous when preserved for a long time in 80% ethanol. Teneral and mature specimens exhibit a similar pattern of dark brown to black spots near the M line and ocelli (Fig 4D–4F).

#### *Anacroneuria polita* (Burmeister 1839)

*Perla polita* Burmeister 1839: 879, description [81]; *Anacroneuria polita*, Zwick 1972: 1163, illustration [69]; Froehlich 2002: 85, illustration [80]; Froehlich 2004: 58, record [67]; Bispo and Froehlich 2004: 98, illustration [60]; Froehlich 2010: 168, catalog [3]; Novaes and Bispo 2014: 459, illustration and picture [86]; Novaes and Bispo 2014: 435, record [42].

(Figs 4J, 4K and 6D)

*Material examined*. **BR, SP, São Miguel Arcanjo**: PECB, Ribeirão de Pedras, bridge, 06.ii.2017, LHA *et al*. col., 1 male. **SP, Sete Barras**: PEI, Saibadela, no data, ASM col., 1 male.

*Morphometric data*. Male (n = 2) forewing length: 9.0–9.5 mm.

*Remarks*. *Anacroneuria polita* is a small and dark species, lighter when stored for a long time in 80% ethanol (Fig 6D). The penial armatures were illustrated by Zwick in 1972 [69], Froehlich in 2002 [80], and Bispo and Froehlich in 2004 [60]. All morphotypes are externally similar, including their body size and color pattern. However, the penial armature of specimens from the Paranapiacaba Mountains are slightly different at the apex and keel when compared to that of the holotype illustrated by Zwick [69] and to that of the specimen illustrated by Froehlich [80].

#### *Anacroneuria subcostalis* Klapálek 1921

*Anacroneuria subcostalis* Klapálek 1921: 326, description [82]; Jewett 1960: 174, record [9]; Froehlich 2002: 86, illustration [80]; Froehlich 2004: 59, male description [67]; Bispo and Froehlich 2004: 99, record [60]; Froehlich 2010: 171, catalog [3]; Baldin *et al*. 2013: 392, record [39]; Gonçalves *et al*. 2017: 147, distribution [43].

(Fig 6E)

*Material examined*. **BR, SP, Iporanga**: PEI, Ribeirão Água Comprida, 09.iii.2004, ASM col., 1 male and 1 female; PEI, Córrego Roda D’Água, 16.iii.2013, LSL col., 1 male and 4 females.

*Morphometric data*. Male (n = 2) forewing length: 10.6–11.0 mm. Female (n = 5) forewing length: 12.0–13.2 mm.

*Remarks*. *Anacroneuria subcostalis* is an easily identified species in the Paranapiacaba Mountains because of its characteristic color pattern and penial armature. This species is mainly white with brown bands on the sides of the pronotum (Fig 6E) and a dark subcostal vein. The center of the head has a yellow spot (Fig 6E). This color pattern is uncommon for species from southeastern Brazil. However, it is often seen in several northern and northeastern species [87, 89, 92].

#### *Anacroneuria tupi* Bispo and Froehlich 2004

**Fig 9:**
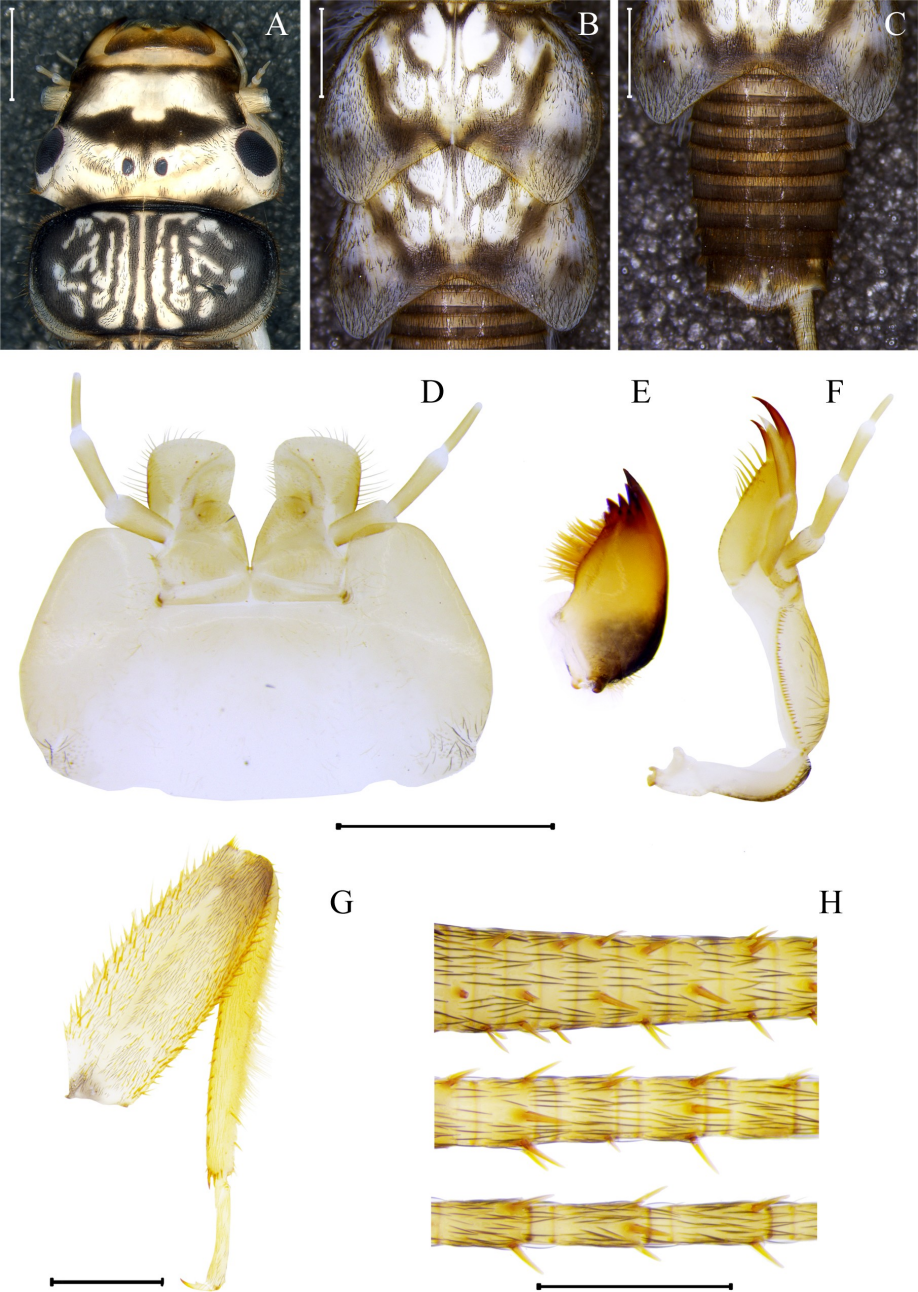
*Anacroneuria tupi* nymph. Head and pronotum (A), mesonotum and metanotum (B), abdomen (C), labium (D), right mandible (E), right maxilla (F), foreleg (G), cercus in proximal, medial and distal views (H). Scales: 1 mm for A–G and 0.5 mm for H.

*Anacroneuria tupi* Bispo and Froehlich 2004: 105, description [60]; Froehlich 2010: 173, catalog [3].

(Figs 4G–4I, 6F and 9A–9H)

*Material examined*. **BR, SP, São Miguel Arcanjo**: PECB, Rio Taquaral, bridge, 09.ix.2006, PCB col., 3 males; PECB, Braço do Rio Taquaral, bridge, 24°03’31”S, 47°59’41”W, 05.ii.2017, LHA *et al*. col., 1 male. **SP, Iporanga**: PEI, Ribeirão Lajeado, 24.ix.1999, PCB and VLCB col., 1 male; PEI, Rio do Carmo, near Córrego do Inferno, 29.ix.2002, ASM col., light trap, 2 males; PEI, Rio do Carmo, bridge, 14.xii.2014, PCB *et al*. col., 3 nymphs; 08.ii.2017, LHA *et al*. col., 1 nymph; 09.ii.2017, LHA *et al*. col., 3 males.

*Morphometric data*. Male (n = 11) forewing length: 10.0–11.2 mm. Nymph (n = 3): head width 2.5–2.7 mm, head length 2.1 mm, pronotum width 2.6–2.8 mm, pronotum length 1.4–1.5 mm, cercal length 6.5 mm, total length 9.2–9.7 mm.

*Description of nymphs*. General dark-brown color in the dorsal view and light-yellow in the ventral view. Head bicolored, labrum brown, frontoclypeus brown with an ochraceous anterior band and a large light-yellow spot in the middle; posterior part of head light-yellow (Fig 9A). Eyes and ocelli black (Fig 9A). Labium light-yellow (Fig 9D); mandible brown with darker base and with five pointed teeth (Fig 9E); maxillae light-yellow (Fig 9F). Pronotum dark-brown with median line and rugosities light-yellow (Fig 9A). Wing pad light-yellow with brown spots (Fig 9B). Foreleg light-yellow; femur covered by sparse bristles and a central dorsal line without setae, anteriorly and posteriorly with high density of thick bristles, and posteriorly with a fringe of hairs (Fig 9G); tibia anteriorly with sparse thick bristles, and posteriorly with a row of thick bristles and a well-developed band of hairs (Fig 9G). Abdomen dark-brown following the pattern of body and with a light spot in the center of the 10th tergum (Fig 9C). Cerci light-yellow with thick bristles, segments differing in shape and size from base to apex (Fig 9H). Thoracic gills: ASC1, AT2, AT3, and PT3.

*Remarks*. The teneral specimens has the same two dark spots on the head as the mature adult specimens (Fig 4G–4I). The nymph also has a head with a characteristic color pattern that is different from all nymphs described so far for the genus.

#### *Kempnyia auberti* Froehlich 1996

*Kempnyia auberti* Froehlich 1996: 117, description [70]; Froehlich 2010: 179, catalog [3].

*Material examined*. **BR, SP, Iporanga**: PEI, Rio Poços Altos, 10.xi.1993, CGF and ASM col., 1 male (Holotype/MZUSP).

*Morphometric data*. Male (n = 1) forewing length: 22 mm.

*Remarks*. The holotype is a large specimen with a brown general color. The penial armature is similar to that of *K*. *neotropica* but presents differences in the membranous areas, especially in the lateral view. These species were separated by Froehlich [70] based mainly on differences in the hammer, body size, and color pattern. As only two specimens of *K*. *auberti* are known, of which only one is deposited in Brazil, it is not possible to assess whether the morphological variability of this species overlap with the variability of *K*. *neotropica*. Therefore, collecting new specimens and obtaining the DNA barcode are fundamental to establish the real limits of *K*. *auberti*.

#### *Kempnyia colossica* (Navás 1934)

**Fig 10:**
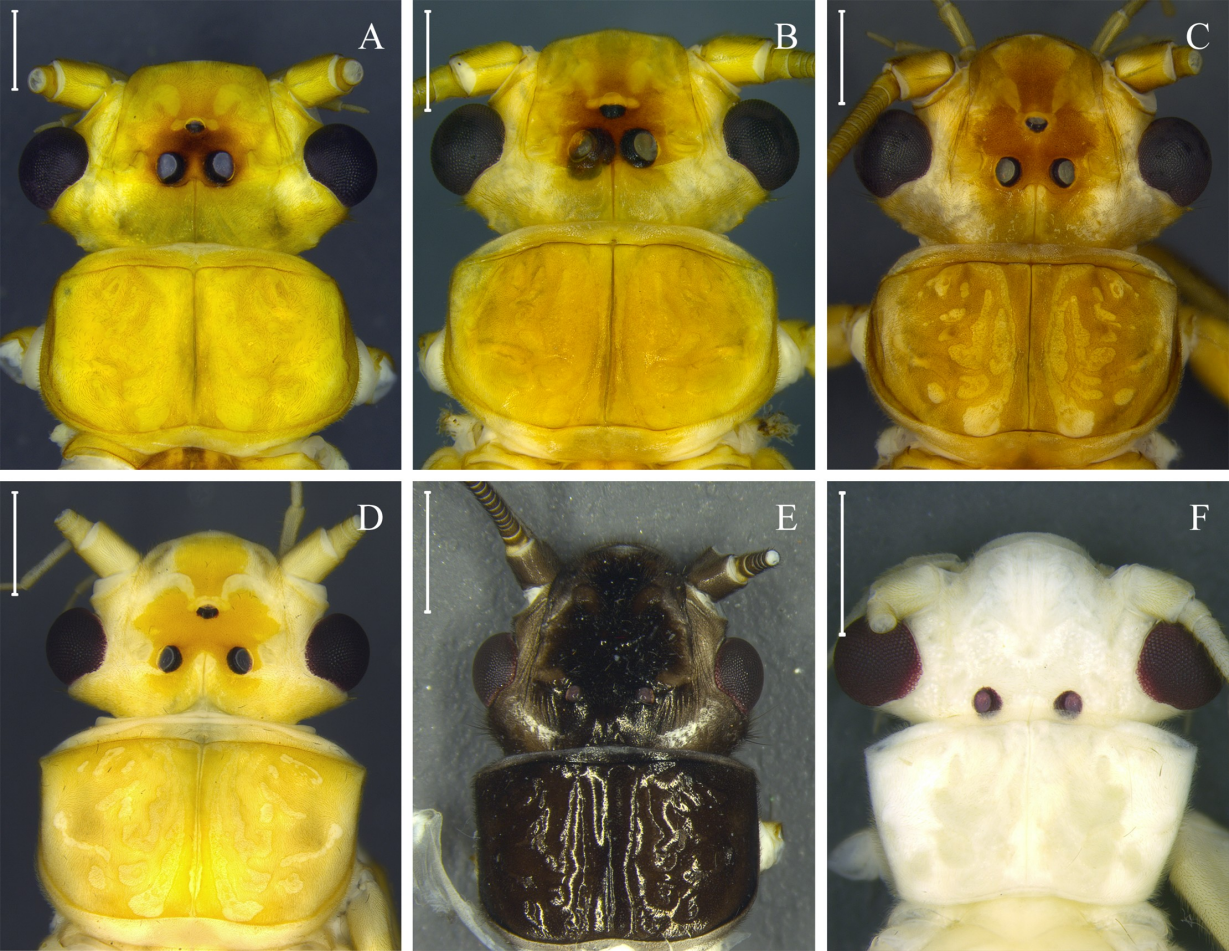
*Kempnyia* and *Macrogynoplax* species. Head and pronotum of a *K*. *colossica* adult male from the Paranapiacaba Mountains (A), PR (B) and SC (C), a *K*. *flava* adult female (D), a *K*. sp. adult female (E) and an *M*. *veneranda* adult male (F). Scale: 1 mm.

*Laeissa colossica* Navás 1934: 22, description [68]; Jewett 1959: 151, illustration [93]; *Kempnyia colossica* Froehlich 1988: 154, illustration [20]; Bispo and Froehlich 2004: 110, record [60]; Froehlich 2010: 180, catalog [3]; Froehlich 2011: 21, record [13]; Bispo *et al*. 2013: 02, nymph description [39]; Novaes and Bispo 2014: 464, illustration and picture [86]; Avelino-Capistrano *et al*. 2014: 330, picture [21]; Gonçalves *et al*. 2017: 147, misidentification [43]. (Fig 10A–10C)

*Material examined*. **BR, PR, Morretes**: Mata Atlântica Park Hotel, 12.i.2012, M.C. Novaes col., 1 male and 1 female. **SC, Grão Pará**: Parque Estadual Serra Furada, Rio CAPEA, 08.i-16.ii.2013, L.C. Pinho col., malaise, 2 males. **SP, São Miguel Arcanjo**: PECB, Ribeirão de Pedras, bridge, 06.ii.2017, LHA *et al*. col., 1 male and 1 nymph. **SP, Iporanga**: PEI, Rio do Carmo, bridge 20.ii.2000, PCB col., 1 male; 14.xii.2014, PCB *et al*. col., 3 males; PEI, Ribeirão Bocaina, 27.vii.2000, PCB and VLCB col.,1 nymph; PEI, Córrego Araçá, 24°19’35”S, 48°25’52”W, 28.x.2002, ASM col., 1 male and 1 female.

*Morphometric data*. Male (n = 6) forewing length: 22.5–28.0 mm. Female (n = 1) forewing length: 35.0 mm.

*Remarks*. Froehlich [20] reported slight variations in color pattern and penial armature between specimens from São Paulo and Santa Catarina states. Here, our molecular analysis revealed a high intraspecific divergence (13.9%), which was the highest among the *Kempnyia* and was close to the lowest recorded interspecific divergence for the genus (15.3%). The methods of delimiting species suggested that what is currently called *K*. *colossica* is two (ABGD_p_) or three (ABGD_r,_ PTP, and bPTP) species (Fig 2). ABGD_p_ indicated one cluster formed by specimens from the Paranapiacaba Mountains (São Paulo State) and Paraná State (intra-cluster divergence <5.6%) and another formed by specimens from Santa Catarina State (intra-cluster divergence <0.5%). ABGD_r_, PTP, and bPTP indicated three clusters, one from each sampled region: the Paranapiacaba Mountains (intra-cluster divergence 0%), Paraná State (<0.6%), and Santa Catarina State (<0.5%). Morphologically, specimens from the Paranapiacaba Mountains and Paraná State are similar to each other and differ slightly from specimens from Santa Catarina State, both in the color pattern (Fig 10A–10C) and small details of the penial armature. In addition, despite the color difference, *K*. *colossica* has similar morphology and low interspecific divergence (8.35%) in relation to *K*. *guassu* Froehlich 1988 [38]. Although our analyses suggest that the *K*. *colossica* complex contains at least two species, specimens from other populations and from *K*. *guassu* need to be evaluated in an integrative way before the real limits to these species can be established.

#### *Kempnyia flava* Klapálek 1916

*Kempnyia flava* Klapálek 1916: 51, description [83]; Jewett 1960: 177, illustration [9]; Zwick 1972: 1171, illustration [69]; Froehlich 1988: 153, illustration [20]; Bispo and Froehlich 2004: 109, record [60]; Froehlich 2010: 180, catalog [3]; Froehlich 2011: 21, record; Gonçalves *et al*. 2017: 147, record [43].

(Fig 10D)

*Material examined*. **BR, SP, São Miguel Arcanjo**: PECB, Rio Bonito, bridge, 24°08'31.6"S, 47°59'41.3"W, 06.ii.2017, LHA *et al*. col., 2 females. **SP, Iporanga**: PEI, Rio do Carmo, bridge, 28.x.1999, PCB and VLCB col., 1 male; 20.ii.2000, PCB, 1 male.

*Morphometric data*. Male (n = 2) forewing length: 12.4–14.0 mm. Female (n = 2) forewing length: 13.9–14.2 mm.

*Remarks*. Species predominantly yellow with characteristic penial armature [20].

#### *Kempnyia neotropica* (Jacobson and Bianchi 1905)

*Perla* (*Perla*) *obscura* Pictet 1841: 28, description [4]; *Perla neotropica* Jacobson and Bianchi 1905: 617, renaming [78]; *Macrogynoplax aterrima* Klapálek 1916: 73, description [83]; *Kempnyia neotropica*, Zwick 1972: 1168, illustration [69]; Zwick 1973: 276, record [84]; Bispo and Froehlich 2004: 2, record [60]; Bispo and Froehlich 2004: 107, record [60]; Bispo and Froehlich 2008: 62, nymph description [33]; Froehlich 2010: 181, catalog [3]; Froehlich 2011: 133, illustration [13]; Froehlich 2011: 22, record [13]; Duarte *et al*. 2014: 89, record [14]; Novaes and Bispo 2014: 464, illustration and picture [86]; Novaes and Bispo 2014: 283, illustration and picture [91]; Novaes and Bispo 2014: 439, record [42]; Novaes *et al*. 2016: 98, record [88]; Gonçalves *et al*. 2017: 147, record [43]. *Kempnyia petersorum* Froehlich 1996: 119, description [70]; Froehlich 2011: 134, illustration [13]; Avelino-Capistrano *et al*. 2014: 331, nymph description [21]. ***New synonym***.

(Fig 5A–5F)

*Material examined*. **BR, SP, São Miguel Arcanjo**: PECB, Ribeirão de Pedras, bridge, 06.ii.2017, LHA *et al*. col., 2 males. **SP, Iporanga**: PEI, 7.ii.1989, E. Trajano col., 1 male (*K*. *petersorum* Holotype/MZUSP); PEI, Rio do Carmo, bridge, 27.ii.1997, ASM col., 1 male; 14.xii.2014, PCB col., 7 females and 1 nymph; 08.ii.2017, LHA *et al*. col., 1 nymph; 09.ii.2017, LHA *et al*. col., 3 males; PEI, Ribeirão Água Comprida, 29.xi.2000, PCB and VLCB col., 1 male.

*Morphometric data*. Male (n = 7) forewing length: 12.0–14.5 mm. Female (n = 7) forewing length: 14.5–15.5 mm.

*Remarks*. Zwick [69] concluded that the penial armature variations of *K*. *obscura* (Pictet 1841) [4] and *K*. *fusca* (Navás 1932) [94] were not sufficient to separate them from *K*. *neotropica*, thereby, synonymizing these three species. Froehlich [70] described *K*. *petersorum* as a new species from the Paranapiacaba Mountains, citing the similarities of its penial armature with *K*. *neotropica*. The main difference was that the types of *K*. *petersorum* have a brown head with lateral light-yellow bands on each side, while in *K*. *neotropica*, the head has a more homogeneous brown color. Since Froehlich [70], several authors have shown the similarities between *K*. *petersorum* and *K*. *neotropica* [21, 33, 60, 86, 91]. In this context, Bispo and Novaes [86, 91], studying Plecoptera from southern Brazil, found both morphotypes: 1) darker brown specimens; and 2) lighter specimens presenting a head with lighter side bands and milky wings. They found no morphological variation that could justify the identification of lighter specimens as *K*. *petersorum*, assuming that they were a variation of *K*. *neotropica*. Avelino-Capistrano *et al*. [21] also found two morphotypes in the Mountains of Southern Brazil, identifying them mainly as *K*. *petersorum* and described the nymph of the species. The described nymph [21] is similar to that of *K*. *neotropica* [33], differing only by the absence of paraproct gills. In the present study, specimens of *K*. *petersorum* (Fig 5D–5F) and *K*. *neotropica* (Fig 5A–5C) formed a single cluster (Fig 2) with a low COI divergence (0–3.6%). The low molecular divergence (<3.6%), results of species delimitation methods (ABGD_p_, ABGD_r_, PTP, and PTP), and high morphological similarity indicated that they are the same species. Therefore, in this study, the two species are considered synonymous. Here, the color pattern observed in *K*. *petersorum* is considered that of teneral *K*. *neotropica*.

#### *Kempnyia* sp. (Fig 10E)

*Material examined*. **BR, SP, Iporanga**: PEI, Rio do Carmo, bridge, 14.xii.2014, PCB *et al*. col., 1 female.

*Morphometric data*. Female (n = 1) forewing length: 13.5 mm.

*Remarks*. As the male adult was not collected, it was not possible to identify the species by name. Either way, it is none of the known *Kempnyia* species from the Paranapiacaba Mountains. The specimen does not have the third ocelli (Fig 10E). In addition, the head is dark (Fig 10E) and the post-frontal line is not visible in the studied specimen.

#### *Macrogynoplax veneranda* Froehlich 1984

*Macrogynoplax veneranda* Froehlich 1984: 39, description [19]; Bispo and Froehlich 2004: 111, record [60]; Gonçalves *et al*. 2019: 109, record [95].

(Fig 10F)

*Material examined*. **BR, SP, Iporanga**: PEI, v.1992, CGF col., 1 female; PEI, Córrego Pedrinhas, 19.ii.2000, ASM col., 1 male; PEI, Córrego Roda D’Água, 11.iii.2013, LSL col., 3 females; 14.xii.2014, PCB col., 1 nymph; 07.ii.2017, LHA *et al*. col., 4 males; PEI, Córrego Cacadinho, 14.iii.2013, LSL col., 1 female; PEI, stream behind the Sede de Pesquisa, 15.iii.2013, LSL col., 1 male; PEI, Córrego do Mirante, 15.xii.2014, PCB col., 1 nymph.

*Morphometric data*. Male (n = 6) forewing length: 14.3–14.6 mm. Female (n = 3) forewing length: 16.9–17.4 mm.

*Remarks*. *Macrogynoplax veneranda* is the only species of the genus found in southeastern Brazil. The live adult is predominantly green; after preservation in ethanol, it lost color over time, first turning yellow and then white (Fig 10F).

## Discussion

In this paper, 15 species of Perlidae were recorded in the Paranapiacaba Mountains, one more species than that reported by Bispo and Froehlich [60]. They studied the fauna of one of the conservation areas of the Paranapiacaba Mountains (PEI). Here, two species, *A*. *debilis* and *A*. *fiorentini*, were recorded for the first time in the Paranapiacaba Mountains; the specimen identified as *A*. *petersi* in Bispo and Froehlich [60] was considered a teneral specimen of *A*. *flintorum* and *K*. *petersorum* was considered a junior synonym of *K*. *neotropica*. The COI was also obtained for 11 species, expanding the database for Brazilian perlids [21, 37, 38].

The COI sequences also made it possible to properly associate all nymphs included in the analysis with their respective adults. Among the species of Perlidae from the Paranapiacaba Mountains, seven have known nymphs, including those associated and described in this study [19, 22, 38, 56–58]. Expanding the knowledge about nymphs is a necessary step since only about 20% of Brazilian species of Perlidae have described nymphs. Rearing nymphs in the laboratory or in the field is a time-consuming method since several species are sensitive to environmental changes [96–98] and, therefore, hard to rear to adulthood [21, 23]. Currently, the use of COI barcode sequences has made this process more efficient and increased the number of known nymphs [21, 37, 38]. Therefore, the expansion of the COI database for Brazilian perlids can enhance the association and description of a greater number of nymphs.

Among the 201 species of stoneflies recorded in Brazil [2], COI sequences for only 22 of them, including our sequences, have been obtained [21, 24, 38]. These studies are focused on Perlidae and have shown intraspecific variation from 0 to 15%. For example, Avelino-Capistrano *et al*. [21, 37] found the following maximum COI intraspecific divergences considering specimens mainly from Bacia do Rio Macaé, Rio de Janeiro State: *Kempnyia colossica*, 15.1%; *K*. *gracilenta* (Enderlein 1909) [5], 11.2%; *K*. *jatim* Froehlich 1988 [20], 7.7%; *K*. *obtusa* Klapálek 1916 [83], 9.6%; and *K*. *varipes* Klapálek 1916 [83], 9.6%. These values are higher than those observed in the present study, where, except for *K*. *colossica* (about 14% divergence), all other species showed intraspecific divergence <4%. These values are compatible with those found by Avelino-Capistrano *et al*. [37], who found 0.2% intraspecific divergence for *K*. *couriae* Avelino-Capistrano, Barbosa and Takyia 2016 [38], and by Almeida *et al*. [24], who, as previously mentioned, found approximately 4% of intraspecific divergence considering individuals from different populations of *A*. *flintorum*.

Our results revealed that there was good congruence between morphological and molecular variation, suggesting that COI intraspecific and interspecific divergence may effectively assist the identification and delimitation of species of Plecoptera, confirming the results of other studies [99–102]. The maximum intraspecific divergence, considering only the specimens collected in the Paranapiacaba Mountains, was <4%. Conversely, it is natural to assume that expanding the collection areas to other regions besides the Paranapiacaba Mountains would increase this divergence. For example, *A*. *flintorum* presented a divergence <1% when considering only the specimens of the Paranapiacaba Mountains and <4% when considering specimens throughout its geographic distribution [24]. This is even clearer for *K*. *colossica*, for which there was no intraspecific divergence for the specimens collected in the Paranapiacaba Mountains. In contrast, when specimens from Paraná State were included, the maximum divergence increased to approximately 6%, and when specimens from Santa Catarina State were included, it increased to approximately 14%.

The intraspecific divergence of *K*. *colossica* found based on specimens from different populations was high, suggesting that it is a complex of cryptic species. As previously mentioned, Avelino-Capistrano *et al*. [38] considered specimens from Rio de Janeiro and São Paulo states and also found similar results, with a maximum intraspecific divergence of approximately 15%. Notably, before molecular approaches, Froehlich in 1988 [68] had already observed slight morphological variations between specimens from São Paulo and Santa Catarina states. This morphological variation was also observed in the present study and was congruent with the molecular variation (Fig 2). The results of the methods of delimiting species based on molecular data (ABGD_p_, ABGD_r_, PTP, and bPTP) (Fig 2) added to the corresponding morphological variation suggested that *K*. *colossica* as currently defined, could be at least two different species. In addition, Avelino-Capistrano *et al*. [38] recorded a lower interspecific COI divergence (8.3%) between *K*. *colossica* and *K*. *guassu* than the highest intraspecific divergence observed for *K*. *colossica* (~15%) [38]. *Kempnyia colossica* and *K*. *guassu* are similar, differing mainly in terms of color, as the former is lighter than the latter. Therefore, the study of a larger number of specimens of *K*. *guassu* and different *K*. *colossica* morphotypes using an integrative approach is necessary to reevaluate the limits of these species.

Although a threshold of 2–3% in COI divergence has commonly been considered for the definition of insect species [61, 102], intraspecific divergence can be quite variable and those >3%, as observed here and in Avelino-Capistrano *et al*. [17], are not uncommon in Plecoptera [99, 103]. Some recent examples include: 1) among five species of *Besdolus* Ricker (Perlodidae), three had intraspecific divergence >3%: *B*. *illyricus* Kovács and Zwick 2008 (3.3%), *B*. *ventralis* (Pictet 1841) (3.3%), and *Besdolus bicolor* (Navás 1909) (8.3%) [53]; 2) among 11 species of *Siphonoperla* Zwick (Chloroperlidae), two had intraspecific divergence >3%: *S*. *hajastanica* (Zhiltzova 1961) (5.1%) and *S*. *torrentium* (Pictet 1841) (6.2%) [54]; 3) among 16 species of western Neartic *Isoperla* Banks (Perlodidae), five had intraspecific divergence >3%: *I*. *adunca* Jewett 1962 (7.7%), *I*. *bifurcata* Szczytko and Stewart 1979 (3.6%), *I*. *marmorata* (Needham and Claassen 1925) (5.1%), *I*. *quinquepunctata* (Banks 1902) (5.1%), and *I*. *mormona* Banks 1920 (5.5%) [100]; 4) among 37 species of *Leuctra* Stephens (Leuctridae) from Switzerland, three had intraspecific divergence >3%: *L*. *armata* Kempny 1899 (3.6%), *L*. *cingulata* Kempny 1899 (5.8%), and *L*. *nigra* (Olivier 1811) (10.6%) [101]; and 5) in a recent study for some species from China, 15% intraspecific divergence was found in *Rhopalopsole sinensis* Yang and Yang 1993 (Leuctridae) [99]. Therefore, data from the literature and the present study revealed that the intraspecific divergence in Plecoptera can vary widely and defining species limits automatically, using only a fixed COI divergence threshold, can overestimate the number of species. However, the high intraspecific divergence found for some species may suggest the existence of cryptic species.

The use of a fixed molecular divergence threshold for the delimitation of species does not consider the sampling effort (number of specimens studied, number of populations studied) and biology of the species (e.g. number of generations per year, the dispersion capacity, etc.), which can affect intraspecific variability [104, 105]. According to Bergsten *et al*. [106], there is a correlation between sampling efforts throughout a geographical range and genetic variations. Basically, when the species are sampled in their entire geographical distribution, the intraspecific divergence tends to increase and interspecific divergence to decrease [106]. In addition, several species of Plecoptera have restricted distributions and occur in low- and medium-order streams in clean and well-oxygenated waters; these conditions are spatially restricted in the landscape and can facilitate population isolation, which could be related to the high intraspecific divergence, or even the presence of cryptic species, as observed for some of the species of Plecoptera [107]. This also could explain the high COI intraspecific divergence of specimens in the *K*. *colossica* complex since these stoneflies only occur in small streams with dense vegetation cover in mountainous areas. Based on the comments above, it is expected that the intraspecific divergence values observed here and in other species studied in Brazil [21, 24, 38] will increase when a greater number of individuals and populations are studied.

In insects, the color may change between newly emerged individuals and those that have their final color [108]. This phenomenon can have taxonomic implications, since it can create confusion when the color pattern is used as one of the criteria for delimitation of species [108]. In Plecoptera, this subject has not received the necessary attention; however, ontogenetic polymorphisms associated with adult color have been suggested for some Brazilian species (e.g. *K*. *neotropica* [21, 91]; *A*. *debilis* and *A*. *patioba* Almeida and Duarte 2017 [89]; and *A*. *flintorum* [24]). Our molecular data reinforces this polymorphism for *A*. *debilis*, *A*. *flintorum*, and *K*. *neotropica*, and, for the first time, reveals it for *A*. *fiorentini*, *A*. *itajaimirim*, *A*. *tupi*, and *A*. *polita* (Fig 4). In general, teneral adults differ from those with their final color, mainly in the head and pronotum with white spots on the lateral areas, rounded pronotum without lateral folds [24], presence of remnants of thoracic gills, and milky wings. Some species of Brazilian Perlidae have been delimited based on these characters, which are associated with teneral specimens (e.g. *Anacroneuria petersi* [80] and *Kempnyia petersorum* [70]). Here, after recognizing the color patterns of teneral specimens, *K*. *petersorum* was considered a teneral form of *K*. *neotropica*.

Currently, more than 500 species of Plecoptera have been recorded in South America [2, 3, 11] and the rate of new species described each year is high, approximately 9 new species/year [1]. The problem is that most of these have been described based on a few individuals, often a single adult male, which makes it difficult to understand the variability within and between species. This problem is especially important when considering genera with a high richness of species, such as *Anacroneuria*, which has more than 330 described species [2], many of which have poorly defined limits. The expansion of the collection effort has allowed the constant discovery of new species and increased knowledge about the morphological variability of known species. Given this, the previously defined limits appear to be insufficient for the definition of several species. In this context, it is important to describe new species and reassess those described so far based on an integrative taxonomy [44–46]; thus, it will be possible to more clearly define the limits proposed for each species.

### Moving forward in South American Perlidae taxonomy

The present study focused on the fauna of the Paranapiacaba Mountains; however, based on the results obtained and analyzing the current knowledge, it is possible to make the following recommendations so that knowledge about South American Perlidae continues to evolve: 1) expand collections and preserve specimens in appropriate ways for molecular analysis, which will enable an increase in the frequency of studies using an integrative approach [44–46]; 2) use molecular tools to associate adults and nymphs, freeing researchers from the need to rear nymphs to adulthood [21, 24, 38]; 3) consider that color may vary depending on the time since emergence and the time for which the specimen was preserved in ethanol (bleaching of specimens occurring over time). Thereby, we recommend discarding color as one of the criteria used to delimit species [24]; 4) define species based on different sources of evidence (e.g. morphology and molecular) and seek to understand the processes of diversification through phylogeographic, phylogenetic and biogeographic approaches.

The delimitation of South American Perlidae species is not a simple task, even using an integrative principle [109]. The high diversity recorded thus far, 457 species of Perlidae in South America [2], and the delimitations based on small details of the penial armature or variation in color have caused difficulties in identification. This is clear from several examples, two of which are cited: 1) *A*. *vistosa* Stark 1995 [28], *A*. *paria* Stark 1999 [110] (both from Amazon Forest, Venezuela), and *A*. *marlieri* Froehlich 2001 [111] (from Amazon Forest, Brazil) have similar penial armature, being distinguish mainly by color pattern; and 2) the Brazilian species *A*. *debilis* [4], *A*. *stanjewetti* Froehlich 2002 [80], *A*. *uyara* Froehlich 2002 [80], *A*. *brandaoi* Bispo and Froehlich 2004 [112], and *A*. *lecoensis* Righi-Cavallaro and Lecci 2013 [40], which differ in body size and in minor details in penial armature, but there are some overlapping characteristics, causing difficulties for identification. Notably, the body size may be affected by the time of emergence and by local environmental factors (e.g. temperature and food availability and quality), therefore, it must also be used with caution in taxonomy of Plecoptera [113, 114]. In addition, it is important to correlate the existing type specimens with newly collected specimens. Unfortunately, several old types have been lost or are in poor condition. Therefore, recovering the previous knowledge about the species, associating the new knowledge that is currently being obtained, and better defining the species limits are essential to improve the understanding of South American Perlidae.

Researchers like C.G. Froehlich and B.P. Stark have built a solid foundation for the knowledge of Perlidae fauna in South America [3, 11]. Through the study of these two researchers [13, 28, 29, 70, 80, 110, 115, 116] and other researchers [38, 39, 43, 60, 86–89, 117–120], the number of species described in the last 30 years has increased by approximately 180% [2]. Currently, as new technologies are becoming increasingly accessible, it is important that this previously acquired knowledge is complemented using an integrative approach. The use of penial armature represented a great advance in the definition of species from South American Perlidae since 1970 [18, 19, 69], and it is now time to change the paradigm by including also other types of data in addition to morphology and moving increasingly towards a more integrative taxonomy. Thus, it will be possible to continue advancing the increasingly solid basis for the knowledge of Perlidae diversity in South America.

## Supporting information

S1 Table Voucher codes. Specimen vouchers with respective identification, collecting local and GenBank accession codes of COI sequences.
(PDF)

S2 Table Kimura-2-parameter (K2P) divergences of COI sequences. (CSV)

S1 Appendix Key identification to male adults of Perlidae from the Paranapiacaba Mountains (modified from Bispo and Froehlich, 2004 [60]) Fw–male forewing length. (PDF)

S1 Fig Molecular analysis. Neighbor-Joining tree modeled by K2P for *mitochondrial cytochrome c oxidase subunit I* (COI) sequences (636 bp) from specimens from the Paranapiacaba Mountains and related stoneflies from PR (red rectangle) and SC (blue rectangle), Brazil. Numbers are bootstrap support. The tree was rooted using a sequence of Gripopterygidae.
(TIF)

S2 Fig Brazilian states. Brazilian states with their respective acronyms and full names.
(TIF)
