# Supplementary material for: Perlidae (Plecoptera) from the Paranapiacaba Mountains, Atlantic Forest, Brazil: Diversity and implications of the integrative approach and teneral specimens on taxonomy
Source: PLoS One. 2020 Dec 10;15(12):e0243393. doi: 10.1371/journal.pone.0243393 (PMC7728281; doi:10.1371/journal.pone.0243393)
Supplement: S1 Table — Specimen vouchers with respective identification, collecting local and GenBank accession codes of COI sequences. (PDF) [file pone.0243393.s001.pdf]

S1 Table. Voucher codes. Specimen vouchers with respective identification, collecting location, and GenBank accession codes of COI sequences.

Species / Specimen life stage	Voucher code	Locality	GenBank Accession
Gripopteryx. sp.. (F)	PPI3	BR, SP, Iporanga, PEI, Roda D'agua	MW293865
A. flintorum Froehlich (F)	PPI8	BR, SP, Iporanga, PEI, Roda D'agua	MG737315.1
A. flintorum Froehlich (F)	PPI9	BR, SP, Iporanga, PEI, Córrego do Mirante	MG737316.1
A. flintorum Froehlich (F)	PPI14	BR, SP, Iporanga, PEI, Rio do Carmo	MG737317.1
A. flintorum Froehlich (M)	LP06	BR, SP, Iporanga, PEI, Rio do Carmo	MG737319.1
A. flintorum Froehlich (N)	LP12	BR, SP, Iporanga, PEI, Córrego do Mirante	MG737320.1
A. flintorum Froehlich (M)	LM16	BR, SP, Apiaí, PETAR, Riacho Furnas	MW293821
A. flintorum Froehlich (M)	LM17	BR, SP, Apiaí, PETAR, Rio Roncador	MW293822
A. polita (Burmeister) (M)	LM12	BR, SP, São Miguel Arcanjo, PECB, Ribeirão de Pedras	MW293828
A. itajaimirim Bispo & Froehlich (F)	PPI15	BR, SP, Iporanga, PEI, Roda D'agua	MW293827
A. itajaimirim Bispo & Froehlich (M)	LM24	BR, SP, Iporanga, PEI, Roda D'agua	MW293823
A. itajaimirim Bispo & Froehlich (M)	LM25	BR, SP, Apiaí, PETAR, Riacho Furnas	MW293824
A. itajaimirim Bispo & Froehlich (M)	LM26	BR, SP, Iporanga, PEI, Rio do Carmo	MW293825
A. itajaimirim Bispo & Froehlich (M)	LM27	BR, SP, Apiaí, PETAR, Riacho Furnas	MW293826
A. tupi Bispo & Froehlich (N)	LP10	BR, SP, Iporanga, PEI, Rio do Carmo	MW293834
A. tupi Bispo & Froehlich (N)	LP11	BR, SP, Iporanga, PEI, Rio do Carmo	MW293835
A. tupi Bispo & Froehlich (N)	LP15	BR, SP, Iporanga, PEI, Rio do Carmo	MW293836
A. tupi Bispo & Froehlich (M)	LM21	BR, SP, São Miguel Arcanjo, PECB, Rio Taquaral	MW293831
A. tupi Bispo & Froehlich (M)	LM22	BR, SP, São Miguel Arcanjo, PECB, Rio Taquaral	MW293832
A. tupi Bispo & Froehlich (M)	LM23	BR, SP, Iporanga, PEI, Rio do Carmo	MW293833
A. subcostalis Klapálek (F)	LM07	BR, SP, Iporanga, PEI, Roda D'agua	MW293829
A. subcostalis Klapálek (F)	LM08	BR, SP, Iporanga, PEI, Roda D'agua	MW293830
A. debilis Pictec (N)	LP14	BR, SP, Iporanga, PEI, Ribeirão Lajeado	MW293820
A. debilis Pictec (M)	LM18	BR, SP, Apiaí, PETAR, Rio Roncador	MW293817
A. debilis Pictec (M)	LM19	BR, SP, Iporanga, PEI, Ribeirão Lajeado	MW293818
A. debilis Pictec (M)	LM20	BR, SP, Apiaí, PETAR, Núcleo Ouro Grosso, Betari	MW293819
M. veneranda Froehlich (N)	LP08	BR, SP, Iporanga, PEI, Roda D'agua	MW293863
M. veneranda Froehlich (N)	LP09	BR, SP, Iporanga, PEI, Córrego do Mirante	MW293864
M. veneranda Froehlich (M)	LM13	BR, SP, Iporanga, PEI, Roda D'agua	MW293861
M. veneranda Froehlich (M)	LM14	BR, SP, Iporanga, PEI, Roda D'agua	MW293862
K. colossica Navás (M)	PPI10	BR, SP, Iporanga, PEI, Rio do Carmo	MW293842

S1 Table. Voucher codes. Specimen vouchers with respective identification, collecting locality and GenBank accession codes of COI sequences.

Species / Specimen life stage	Voucher code	Locality	GenBank Accession
K. colossica Navás (M)	PPI11	BR, SP, Iporanga, PEI, Rio do Carmo	MW293843
K. colossica Navás (M)	LP05	BR, SP, Iporanga, PEI, Rio do Carmo	MW293841
K. colossica Navás (M)	LL14	BR, PR, Morretes, Mata Atlântica Park Hotel	MW293839
K. colossica Navás (F)	LL16	BR, PR, Morretes, Mata Atlântica Park Hotel	MW293840
K. flava Klapálek (F)	LM34	BR, SP, São Miguel Arcanjo, PECB, Rio Bonito	MW293845
K. flava Klapálek (F)	LM35	BR, SP, São Miguel Arcanjo, PECB, Rio Bonito	MW293846
K. neotropica (Jacobson & Bianchi) (F)	PPI4	BR, SP, Iporanga, PEI, Rio do Carmo	MW293855
K. neotropica (Jacobson & Bianchi) (F)	PPI12	BR, SP, Iporanga, PEI, Rio do Carmo	MW293853
K. neotropica (Jacobson & Bianchi) (F)	PPI13	BR, SP, Iporanga, PEI, Rio do Carmo	MW293854
K. neotropica (Jacobson & Bianchi) (N)	LP01	BR, SP, Iporanga, PEI, Rio do Carmo	MW293848
K. neotropica (Jacobson & Bianchi) (F)	LP02	BR, SP, Iporanga, PEI, Rio do Carmo	MW293849
K. neotropica (Jacobson & Bianchi) (F)	LP03	BR, SP, Iporanga, PEI, Rio do Carmo	MW293850
K. neotropica (Jacobson & Bianchi) (F)	LP04	BR, SP, Iporanga, PEI, Rio do Carmo	MW293851
K. neotropica (Jacobson & Bianchi) (F)	LP07	BR, SP, Iporanga, PEI, Rio do Carmo	MW293852
K. petersorum Froehlich (M)	LM28	BR, SP, Iporanga, PEI, Rio do Carmo	MW293856
K. petersorum Froehlich (M)	LM29	BR, SP, Iporanga, PEI, Rio do Carmo	MW293857
K. neotropica (Jacobson & Bianchi) (M)	LM30	BR, SP, São Miguel Arcanjo, PECB, Ribeirão de Pedras	MW293858
K. petersorum Froehlich (M)	LM31	BR, SP, Iporanga, PEI, Rio do Carmo	MW293859
K. petersorum Froehlich (M)	LM33	BR, SP, Iporanga, PEI, Rio do Carmo	MW293860
K. neotropica (Jacobson & Bianchi) (N)	LM39	BR, SP, Iporanga, PEI, Rio do Carmo	MW293847

(M), (F) and (N) are respectively male, female and nymph
