# Supplementary material for: Perlidae (Plecoptera) from the Paranapiacaba Mountains, Atlantic Forest, Brazil: Diversity and implications of the integrative approach and teneral specimens on taxonomy
Source: PLoS One. 2020 Dec 10;15(12):e0243393. doi: 10.1371/journal.pone.0243393 (PMC7728281; doi:10.1371/journal.pone.0243393)
Supplement: S1 Appendix — (PDF) [file pone.0243393.s003.pdf]

Key identification to male adults of Perlidae from the Paranapiacaba Mountains (modified from Bispo and Froehlich, 2004 [1]). Fw – ♂ Forewing length.

1. Hammer projecting, conical, if short, then rounded. Postfrontal line V-shaped. 2

– Hammer otherwise. Postfrontal line W-shaped 10

2. Hammer short and rounded (Fig 15 in Bispo and Froehlich, 2004 [1]) (Fw 16–17 mm) . . . . *Anacroneuria iporanga*

– Hammer conical 3

3. Penial armature without vesicles (Figs 3–4 in Froehlich, 2004 [2]) (Fw 10.9–11.3 mm) . . . . *Anacroneuria boraceiensis*

– Ventral vesicles present 4

4. Frons with a pair of lateral spots from M-line to the level of ocelli/scars 5

– Otherwise 6

5. Pair of dark spots curved (Fig 6F). Penial armature with apex tilted dorsally (Fig 30 in Bispo and Froehlich, 2004 [1]) (Fw 10–11.2 mm) . . . . *Anacroneuria tupi*

– Pair of dark spots tilted and non-curved (Fig 8A). Penial armature with apex non-tilted dorsally (Fig8E) (Fw ~11 mm) . . . . *Anacroneuria fiorentini*

6. Hooks relatively far from ventral vesicles (Figs 8–10 in Bispo and Froehlich, 2004 [1]) (Fw 10–11.5 mm) . . . . *Anacroneuria flintorum*

– Hooks touching or near from ventral vesicles 7

7. General color yellowish, with a pair of dark spots on the side of the pronotum (Fig 6E), forewings with a distal colorless window (Fw 10.6–11 mm) . . . *Anacroneuria subcostalis*

– Otherwise 8

8. Penial armature subparallel in dorsal/ventral views, a little constricted at middle (Fig 21–22 in Bispo and Froehlich, 2004 [1]) (Fw 10.8–11.5 mm) . . *Anacroneuria itajaimirim*

– Penial armature mainly conical in dorsal/ventral views 9

9. Penial armature with tilted apex in lateral view (Fig 7E). Keel transverse in dorsal view (Fig 7D) (Fw 10–11.4 mm) . . . . *Anacroneuria debilis*

– Penial armature with non-tilted apex in lateral view (Fig 3 in Bispo and Froehlich, 2004 [1]). Keel as two parallel lines in dorsal view (Fig 1 in Bispo and Froehlich, 2004 [1]) (Fw 9–9.5 mm) . . . . *Anacroneuria polita*

10. General color greenish in life, pale yellowish to white in alcohol (Fig 10F). Eyes with medial less pigmented area (Fig 10F) (Fw 14.3–14.6 mm) . . *Macrogynoplax veneranda*

– General color variable. Eyes without medial less pigmented area 11

11. Penial membrane with spiny pads….. 12

– Penial membrane without spiny pads 13

12. Subgenital plate short and straight (Fig 2 in Froehlich, 1996 [3]). Forewing longer than 17 mm (Fw 18–22 mm) . . . . *Kempnyia auberti*

– Subgenital plate long and rounded (Fig 10 in in Froehlich, 1996 [3]). Forewing shorter than 16 mm (Fw 12–15 mm) . . . . *Kempnyia neotropica*

13. Large size species, forewing longer than 20 mm (Fw 22–28 mm) . . . . *Kempnyia colossica*

– Medium size species, forewing shorter than 16 mm (Fw 12.4–14 mm) . . . . *Kempnyia flava*

References

1. Bispo PC, Froehlich CG. Perlidae (Plecoptera) from Intervales State Park, São Paulo State, Southeastern Brazil, with descriptions of new species. Aquat Insects. 2004;26: 97–113. doi:10.1080/01650420412331325837

2. Froehlich CG. *Anacroneuria* (Plecoptera and Perlidae) from the Boracéia and Biological Station and São Paulo State and Brazil. Aquat Insects. 2004;26: 53–63.

3. Froehlich CG. Two new species of *Kempnyia* from southern Brazil (Plecoptera:

Perlidae). Mitteilungen Schweizerische Entomol Gesellschaft. 1996;69: 117–120.
