# Supplementary figures and images for: Perlidae (Plecoptera) from the Paranapiacaba Mountains, Atlantic Forest, Brazil: Diversity and implications of the integrative approach and teneral specimens on taxonomy
Source: PLoS One. 2020 Dec 10;15(12):e0243393. doi: 10.1371/journal.pone.0243393 (PMC7728281; doi:10.1371/journal.pone.0243393)

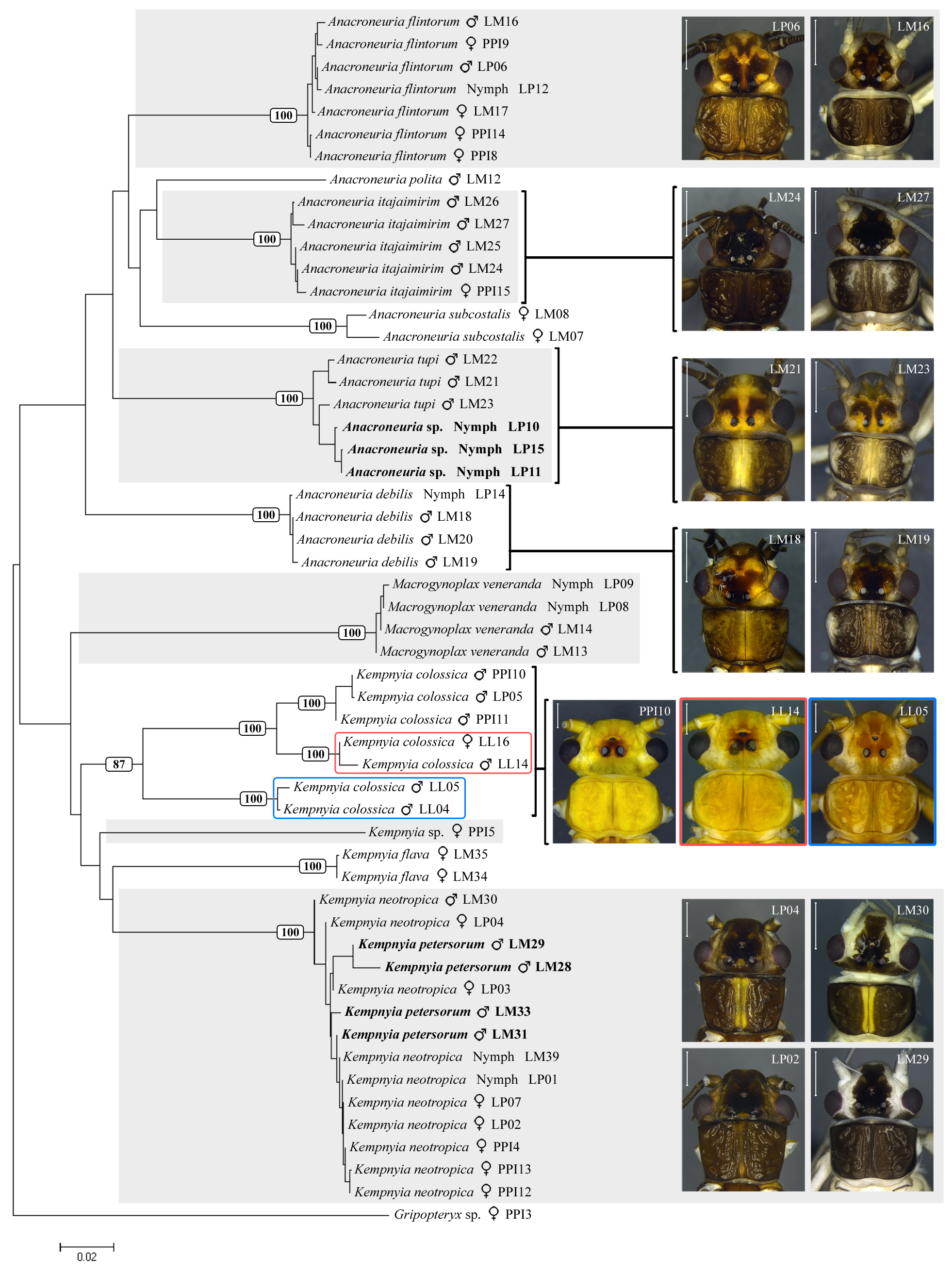

Supplement: S1 Fig — Neighbor-Joining tree modeled by K2P for mitochondrial cytochrome c oxidase subunit I (COI) sequences (636 bp) from specimens from the Paranapiacaba Mountains and related stoneflies from PR (red rectangle) and SC (blue rectangle), Brazil. Numbers are bootstrap support. The tree was rooted using a sequence of Gripopterygidae. (TIF) [file pone.0243393.s004.tif]

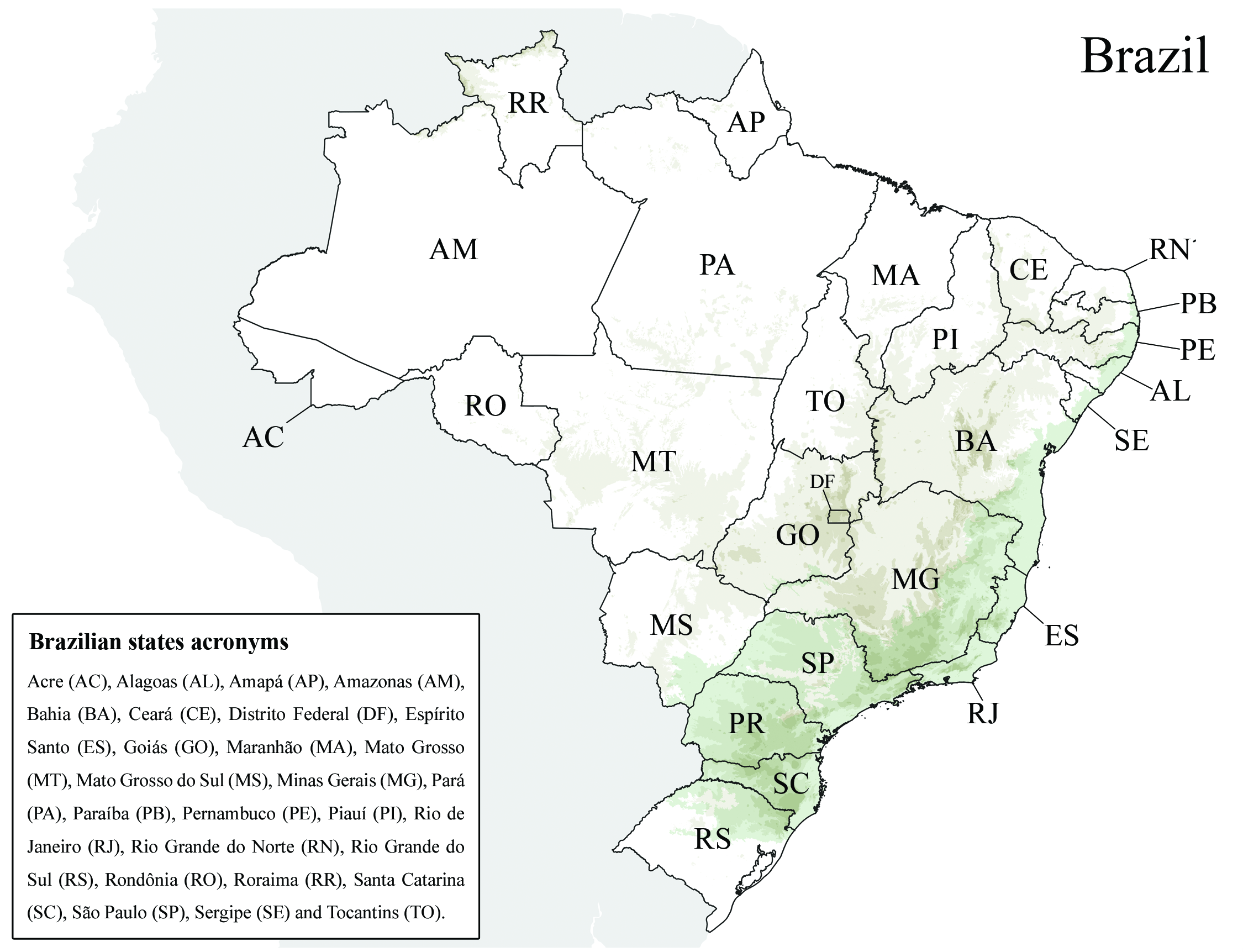

Supplement: S2 Fig — Brazilian states with their respective acronyms and full names. (TIF) [file pone.0243393.s005.tif]
